# A Rhodopsin-Guanylyl Cyclase Gene Fusion Functions in Visual Perception in a Fungus

**DOI:** 10.1016/j.cub.2014.04.009

**Published:** 2014-06-02

**Authors:** Gabriela M. Avelar, Robert I. Schumacher, Paulo A. Zaini, Guy Leonard, Thomas A. Richards, Suely L. Gomes

**Affiliations:** 1 Departamento de Bioquímica, Instituto de Química, Universidade de São Paulo, São Paulo 05508-000, Brazil; 2 Biosciences, University of Exeter, Geoffrey Pope Building, Stocker Road, Exeter EX4 4QD, UK

## Abstract

Sensing light is the fundamental property of visual systems, with vision in animals being based almost exclusively on opsin photopigments [1]. Rhodopsin also acts as a photoreceptor linked to phototaxis in green algae [2, 3] and has been implicated by chemical means as a light sensor in the flagellated swimming zoospores of the fungus *Allomyces reticulatus* [4]; however, the signaling mechanism in these fungi remains unknown. Here we use a combination of genome sequencing and molecular inhibition experiments with light-sensing phenotype studies to examine the signaling pathway involved in visual perception in the closely related fungus *Blastocladiella emersonii.* Our data show that in these fungi, light perception is accomplished by the function of a novel gene fusion (*BeGC1*) of a type I (microbial) rhodopsin domain and guanylyl cyclase catalytic domain. Photobleaching of rhodopsin function prevents accumulation of cGMP levels and phototaxis of fungal zoospores exposed to green light, whereas inhibition of guanylyl cyclase activity negatively affects fungal phototaxis. Immunofluorescence microscopy localizes the BeGC1 protein to the external surface of the zoospore eyespot positioned close to the base of the swimming flagellum [4, 5], demonstrating this is a photoreceptive organelle composed of lipid droplets. Taken together, these data indicate that Blastocladiomycota fungi have a cGMP signaling pathway involved in phototaxis similar to the vertebrate vision-signaling cascade but composed of protein domain components arranged as a novel gene fusion architecture and of distant evolutionary ancestry to type II rhodopsins of animals.

## Highlights

• A rhodopsin-guanylate cyclase gene fusion is involved in *B. emersonii* phototaxis
• The rhodopsin fusion protein BeGC1 is localized to the zoospore eyespot apparatus
• Endogenous retinal substitution by retinalA1 reconstitutes green light phototaxis
• Zoospore phototaxis uses cGMP as a second messenger similar to vertebrate vision

## Teaser

Avelar et al. use genome sequencing, molecular inhibition, and light-sensing phenotype experiments, combined with immunolocalization data, to show that a type I rhodopsin-guanylyl cyclase fusion protein localizes to the “eyespot” and is involved in green light phototaxis in zoospores of the Blastocladiomycete fungus *Blastocladiella emersonii*.

## Results and Discussion

**Figure 1:**
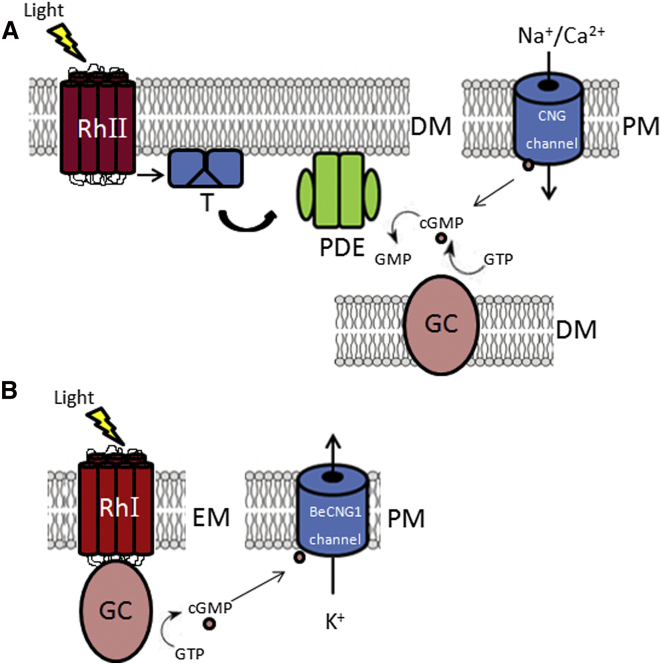
Schematic Models of the Signaling Pathway of Vertebrate Rod Photoreceptor and *Blastocladiella* Zoospore Phototaxis (A) In the vertebrate visual signaling pathway, photoexcitation of the G protein-coupled rhodopsin receptor leads to activation of rod and cone heterotrimeric G protein transducin complex stimulating hydrolysis of cGMP [6]. The decrease in cGMP concentration leads to closure of cGMP-gated (CNG) channels, blockage of Na^+^ influx, and hyperpolarization of photoreceptor plasma membrane, leading to transmission of signal through synapses [6]. Reduction of CNG channel activity blocks Ca^2+^ influx, decreasing cytoplasmic calcium concentration in retinal photoreceptor outer segments (ROS) and leading to activation of guanylyl cyclases (ROS-GCs) by the now Ca^2+^-free GC-activating proteins (GCAPs) through acceleration of ROS-GC dimerization, thus restoring cGMP levels [7, 8]. (B) In the *B. emersonii* zoospore phototaxis transduction pathway, photoisomerization of rhodopsin in BeGC1 activates guanylyl cyclase activity, leading to the synthesis of cGMP from GTP. Cyclic GMP opens K^+^-selective BeCNG1 channels, thereby causing hyperpolarization of the plasma membrane. A putative opening of voltage-activated calcium channels could produce elevation of [Ca^2+^], which would interact with the flagellum altering the flagellar beat, as in *Arbacia* sperm [9]. RhI, type I rhodopsin; RhII, type II rhodopsin; GC, guanylyl cyclase; T, transducin; PDE, phosphodiesterase; EM, eyespot membrane; PM, plasma membrane; DM, disk membrane. See also Figure S4.

Cyclic GMP is an important signaling molecule controlling a large spectrum of physiological responses in eukaryotes. In vertebrates, for example, this system functions with photoreceptors in visual perception (Figure 1A) [6–8]. It is unclear what aspects of this visual perception system are present in other eukaryotes. The fungus *Allomyces reticulatus* forms swimming zoospores and has been suggested to use rhodopsin-mediated signaling to initiate phototaxis [4]. Nevertheless, how the light signal is transmitted to direct flagellar beating is unknown. Publicly available fungal genomes, mostly from ascomycetes and basidiomycetes (Dikarya), demonstrate that these fungi encode opsins, phytochromes, and cryptochromes [10]. However, all of these fungi lack a motile life cycle stage powered by a flagellum, and therefore phototaxis is not observed. *Blastocladiella emersonii* is a close relative of *Allomyces*, branching below the Dikarya and Glomeromycota fungi [11]. Nearly four decades ago, it was demonstrated that the presence of cGMP along with guanylyl cyclase and cGMP phosphodiesterase activities correlated with the completion of *Blastocladiella* sporulation stage during biogenesis of flagellated zoospores [12–14]. These reports indicate the presence of a cGMP signaling pathway, an observation supported by the identification of cDNAs encoding putative guanylyl cyclases and a cGMP phosphodiesterase in *Blastocladiella* transcriptome [15, 16]. In contrast the cGMP pathway appears to be absent in all Dikarya fungi [17].

### Genome Sequencing Data Reveal a Novel Guanylyl Cyclase in *B. emersonii*

To identify *Blastocladiella* cGMP signaling pathway, we sequenced the genome using second-generation sequencing methods. The genome data allowed us to identify the complete nucleotide sequence of a novel guanylyl cyclase-encoding gene (*BeGC1*) with a unique protein domain architecture containing a C-terminal GC catalytic domain and an N-terminal rhodopsin domain, representing a novel gene fusion (Figure S1 available online). We confirmed that this gene architecture is transcribed as a single gene using 5′ rapid amplification of cDNA ends (RACE) data (Figure S1), while quantitative RT-PCR (qRT-PCR) indicates that *BeGC1* transcript is highly expressed in late sporulation cells, during zoospore biogenesis [16]. The *BeGC1* gene is the only rhodopsin found in the draft assembly of *Blastocladiella* genome, with only one melanopsin also being present in the assembled genome. Melanopsin is a photosensitive protein involved in regulating circadian rhythms and other nonvisual reponses to light, with maximum sensitivities near to 480 nm (blue light) [18]. Additional searches of the Blastocladiomycota *Allomyces macrogynus* and *Catenaria anguillulae* genome assemblies confirmed that these fungi also possess the rhodopsin-guanylyl cyclase gene fusion, but with four recent duplications of the *BeGC1* ortholog in *Allomyces* genome and a fission and/or loss of the type I rhodopsin domain in one of these duplication forms (Figure S2); the phenomenon of domain loss and/or fission has been observed frequently in fungi [19]. Orthologs of the *BeGC1* gene were unidentified in all other fungal genome data sets searched (Table S1), including flagellated fungi (checked June 2013).

**Figure 2:**
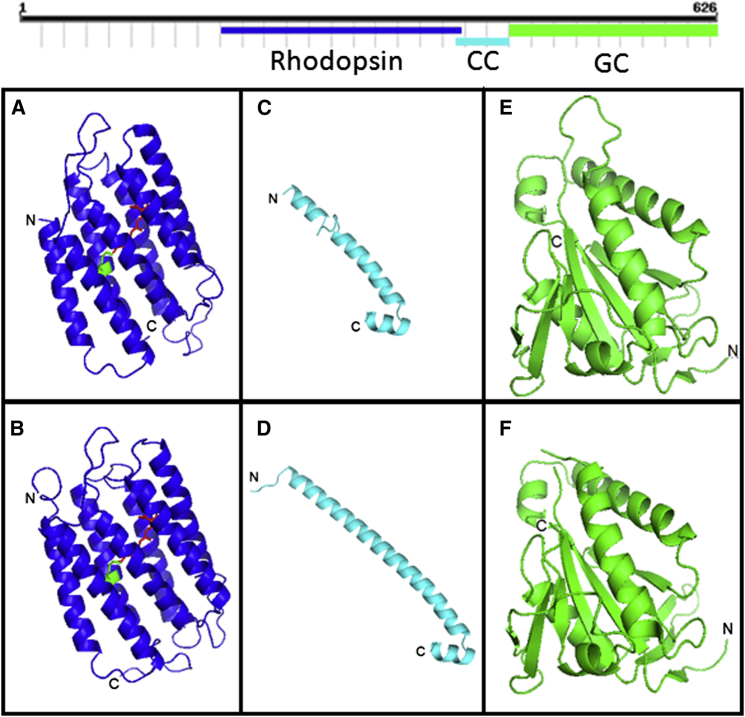
Structural Features of BeGC1 Protein Domains Constructed by Swiss-Model Homology-Based Approach (A and B) The BeGC1 rhodopsin domain structure in (A) is based on the crystal structure of *Halobacterium salinarum* type I rhodopsin [20] shown in (B), with the retinal denoted in red and the lysine of the Shiff base in green. (C and D) The structure of the coiled-coil domain, which links the rhodopsin domain to the GC domain on BeGC1, in (C) is based on the crystal structure of *Rattus norvegicus* soluble guanylyl cyclase CC domain [21] shown in (D). (E and F) The structure of BeGC1 guanylyl cyclase catalytic domain in (E) is based on the crystal structure of the catalytic domain of soluble guanylyl cyclase CYG12 from *Chlamydomonas reinhardtii* [22] shown in (F). See also Figures S1–S3.

The predicted BeGC1 amino acid sequence (626 residues, calculated 68 kDa) demonstrated that residues that putatively interact with the chromophore retinal are conserved, including the lysine known to form a Shiff base (Figure S1). Interestingly, the *Blastocladiella* genome contains the genes necessary for carotenoid biosynthesis (bifunctional lycopene cyclase/phytoene synthase, phytoene dehydrogenase, and carotenoid dioxygenase). Analysis of the C-terminal portion of BeGC1 showed high similarity (65%) to the GC domain of a retinal guanylyl cyclase from *Aedes aegypt* with amino acids that distinguish guanylyl from adenylyl cyclases present (Figure S1). Using homology-based 3D structure modeling, we identified a third protein module: a coiled-coil (CC) domain linking the rhodopsin and GC domains, encompassing 47 amino acids, and with high structural similarity to the CC domain found in all mammalian guanylyl cyclases (Figure 2), a feature shown to be important in regulation and signaling of mammalian guanylyl cyclases [21, 23]. Its function is to prevent constitutive activation of GCs and transmit the activating signal to the catalytic domain [21, 24].

### Phylogenetic Analysis of BeGC1 Guanylyl Cyclase and Rhodopsin Domains

To investigate the evolutionary ancestry of the BeGC1 protein, we conducted phylogenetic analysis of the component domains. The guanylyl cyclase domain phylogeny demonstrates that the Blastocladiomycota gene fusion branches with an unfused putative GC domain homolog from the Chytridiomycota *Gonapodya prolifera* with strong support (Figure S2). The rest of the tree includes a collection of eukaryotic algae, opisthokonts (e.g., animals and fungi), and a sequence from the Amoebozoan *Dictyostelium discoideum*. The patchy taxon distribution and low tree resolution make it difficult to identify the evolutionary ancestry of this domain.

Rhodopsins are seven-transmembrane α helix membrane proteins with a retinal cofactor and have been identified in prokaryotes and eukaryotes. Rhodopsins are classified into two groups, type I and type II, which are also named microbial and metazoan rhodopsins, respectively [25]. There is little sequence identity between type I and type II rhodopsins, making it difficult to align these genes for phylogenetic analysis, although ancestral state reconstruction and analysis of structural similarities have demonstrated that these two gene groups are distantly related [26, 27].

The rhodopsin phylogenetic analysis was restricted to a subsection of the gene family in order to improve tree resolution, demonstrating that Blastocladiomycota rhodopsins branch with type I rhodopsins. The phylogeny showed a very patchy taxon distribution, including prokaryotic sequences, environmental sequences, eukaryotic algae, the choanoflagellate *Salpingoeca rosetta*, and some Dikarya fungi. The Dikarya rhodopsin sequences branch separately from the BeGC1 cluster as a distant paralog (Figure S3). The phylogenetic resolution was poor with Blastocladiomycota sequences branching within a cluster composed of the protist *Salpingoeca* and eukaryotic algae, making it difficult to pinpoint the ancestry of the rhodopsin domain. However, these results demonstrate that the Blastocladiomycota type I rhodopsin-like domain is of distant evolutionary derivation to the type II rhodopsins of animal vision.

### Rhodopsin-Guanylyl Cyclase Activity Is Essential for Phototaxis in *B. emersonii*

**Figure 3:**
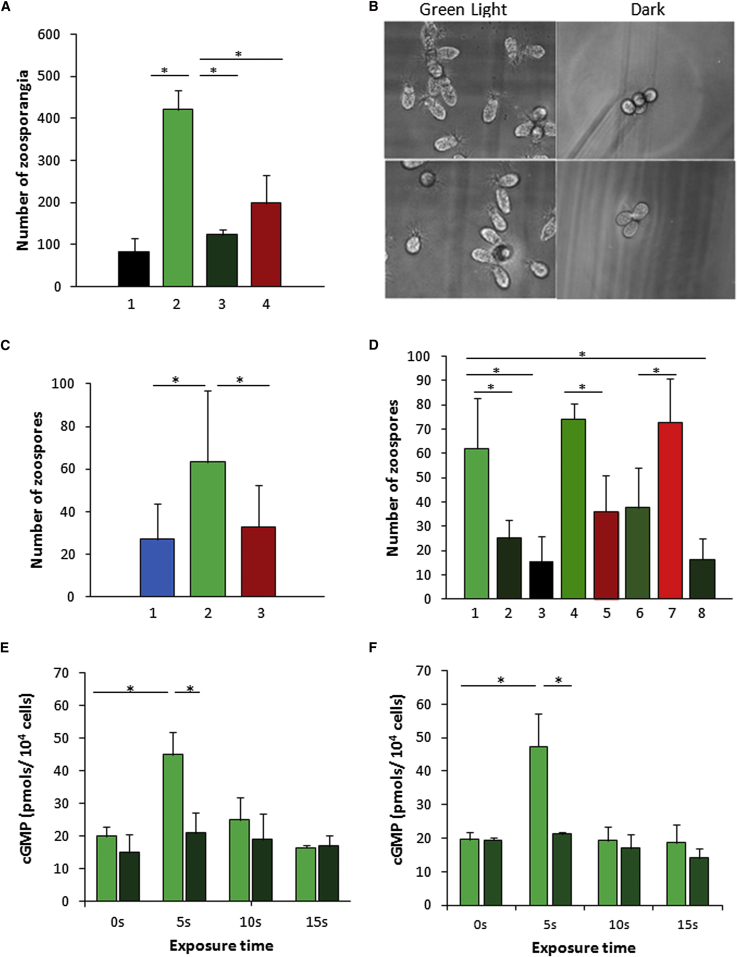
Phototaxis of *B. emersonii* Zoospores Involves Rhodopsin and Guanylyl Cyclase Activity (A and B) Data of phototaxis assays in agar plates. The resulting vegetative cells found in the region of the plates exposed (column 2) or not (column 1) to green light (522 ± 17 nm; 4.4 mW/cm^2^) and preincubated (column 3) or not (column 2) with 500 μM hydroxylamine (HA) were visualized under a light microscope and cells were counted (A) and photographed (B). Vegetative cells were also counted in plates exposed to red light (633 ± 13 nm; 4.4 mW/cm^2^; column 4). Results are mean values of three biological replicates. (C) Phototaxis in microfluidic chamber. Data are from zoospores exposed to blue light (465 ± 25 nm; 67 μW/cm^2^; column 1), zoospores exposed to green light (565 ± 25 nm; 55 μW/cm^2^; column 2) and zoospores exposed to red light (620 ± 30 nm; 35 μW/cm^2^; column 3). (D) Phototaxis in microfluidic chamber with zoospores from growth with norflurazon. Zoospores from growth without (column 1) or with 10 μM (column 2) or 50 μM (column 3) norflurazon exposed to green light, zoospores from growth with 50 μM norflurazon reconstituted with 5 μM retinalA1 exposed to green light (565 ± 25 nm; 55 μW/cm^2^; column 4) or red light (620 ± 30 nm; 35 μW/cm^2^; column 5) or reconstituted with 5 μM retinalA2 exposed to green light (565 ± 25 nm; 55 μW/cm^2^; column 6) or red light (620 ± 30 nm; 35 μW/cm^2^; column 7), and zoospores from growth without norflurazon incubated with 10 μM of GC inhibitor LY83583 exposed to green light (565 ± 25 nm; 55 μW/cm^2^; column 8) are shown. Results are mean values of three biological replicates. (E) Changes in intracellular cGMP levels in zoospores upon green light irradiation. Levels of cGMP were determined before and after different times of zoospore exposure to green light (522 ± 17 nm; 4.4 mW/cm^2^) and in the absence (green rectangles) or presence (dark green rectangles) of 500 μM hydroxylamine. (F) Changes in cGMP levels after different times of irradiation with green light (522 ± 17 nm; 4.4 mW/cm^2^) of zoospores obtained in the presence of 50 μM norflurazon and incubated (green rectangles) or not incubated (dark green rectangles) with 5 μM retinalA1 to restore phototactic capacity. Data are mean values of three independent replicates. Error bars indicate the SE. Asterisks denote significant differences at ^∗^p < 0.05.

For identification of the function of BeGC1, *Blastocladiella* zoospores were exposed to a green light beam source (522 nm) similar to the wavelength at which *Allomyces* zoospores presented their peak (536 nm) phototaxis behavior [4]. *Blastocladiella* zoospores were inoculated in growth media agar plates in a position opposite the light beam. The fraction of zoospores germinated at the light source were counted and compared to the numbers obtained in control plates, which were not exposed to light. These experiments demonstrated a 5-fold increase in zoosporangia under the light source relative to control plates (Figures 3A and 3B).

As a reliable gene knockout protocol is not available for *Blastocladiella*, to test whether rhodopsin is involved in zoospores phototaxis, we used a photobleaching protocol [28] to suppress the rhodopsin function by pre-exposing zoospores to hydroxylamine and green light before performing phototaxis assays at a concentration that did not affect zoospore swimming capacity. A 4-fold reduction in zoosporangia colonization was observed within the light exposed region (Figure 3A).

To check the influence of light wavelength in phototaxis, we conducted the same experiment using a red light source (633 nm), which resulted in less than half the number of zoosporangia colonizing the illuminated region (Figure 3A), demonstrating a preference for green light consistent with results for *Allomyces* phototaxis [4].

To confirm green light phototaxis in *Blastocladiella*, we used direct microscope observations to investigate the movement of zoospores along a microfluidic glass chamber. Zoospores were inoculated at one side of the chamber, and their accumulation was observed under a light microscope in the area opposite to the inoculum, illuminated with light of different wavelengths. Zoospores in this region of the chamber were counted before and after 10 min of illumination, and the numbers indicated that green light is about 2-fold more efficient at promoting phototaxis than is blue light or red light (Figure 3C).

To further establish the selectivity of zoospore phototaxis, we inhibited carotenogenis by growing *Blastocladiella* for three generations in the presence of the inhibitor norflurazon [29]. Treated zoospores demonstrated normal swimming but were incapable of performing phototaxis. However, after incubation with retinalA1, zoospore phototaxis was restored, with green light again being the preferential stimulus compared to red light (Figure 3D). In contrast, when zoospores were incubated with retinalA2, phototaxis was also restored, but red light was the preferential stimulus (Figure 3D), consistent with data demonstrating that retinalA2 serves as a chromophore in red-shifted visual pigments [30]. These data also demonstrate that the rhodopsin is acting as the primary light sensor in our experimental conditions as this shift from green light to red light sensitivity with the addition of retinalA2 is characteristic of rhodopsin rather than melanopsin function [30].

To investigate whether guanylyl cyclase activity is involved in zoospore phototaxis, we determined the intracellular cGMP levels in zoospores exposed to green light for different periods of time using a competitive immunoassay that permits the quantitative determination of cGMP. The levels of cGMP showed a rapid and short-lived increase when zoospores were exposed to green light (Figure 3E), indicating that GC activity in zoospores peaks by 5 s after green light exposure. To check whether rhodopsin function is linked to GC activation in response to green light, we repeated the experiment with zoospores that had undergone photobleaching [28]. In this experiment no increase in GC activity was coupled with green light exposure (Figure 3E), demonstrating that light activation of rhodopsin is linked to GC activation. The levels of cGMP were also investigated in phototaxis experiments with zoospores obtained from cells grown in the presence of norflurazon and incubated with retinalA1 or not incubated. In the presence of retinalA1 and green light, cGMP levels increased significantly upon irradiation, whereas without retinalA1 no increase was observed (Figure 3F). These results indicate that a retinylidene protein is necessary for triggering changes in cGMP levels during green light exposure. We also analyzed phototaxis in *Blastocladiella* zoospores incubated with the guanylyl cyclase inhibitor LY83583 [31] at a concentration at which no effect on completion of the life cycle or on zoospore swimming is observed. The number of zoospores present in the illuminated region was 3.5-fold lower in the presence of LY83583 than in its absence, consistent with GC activity in phototaxis (Figure 3D).

### BeGC1 Is Localized to the Zoospore “Eyespot” Apparatus

**Figure 4:**
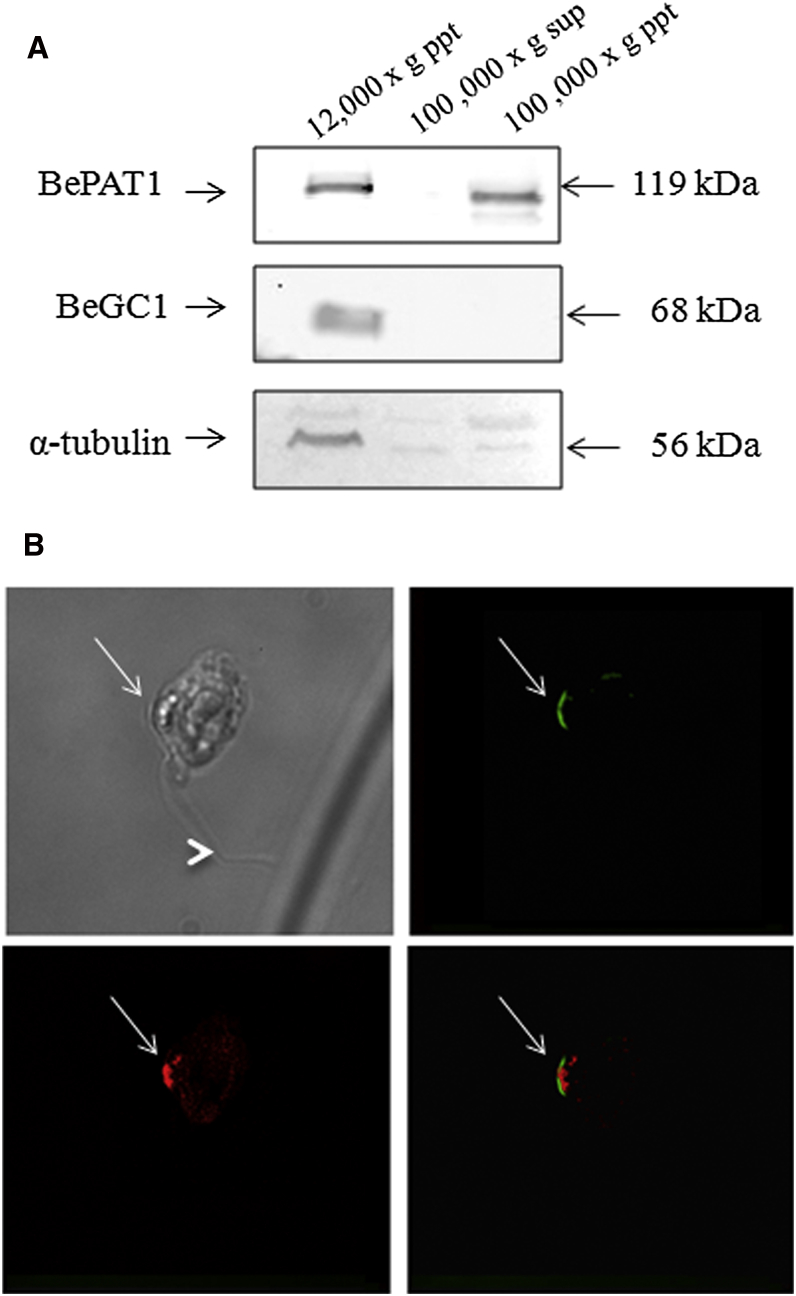
Subcellular Localization of BeGC1 Protein (A) Western blot analysis of subcellular fractions of zoospore lysates obtained by differential centrifugation, as described in the Supplemental Experimental Procedures. Fractions were resolved through SDS-PAGE followed by western blotting and were developed using rabbit antisera against BeGC1, BePAT1, and α-tubulin, as well as the fluorescent CF680 Goat anti-rabbit IgG as a secondary antibody. The bound complexes were detected using the Odyssey Infrared Imaging System. (B) Localization of BeGC1 by immunofluorescence microscopy. Zoospores were fixed with 4% p-formaldehyde and 1% calcium chloride, permeabilized with PBS containing 0.1% Triton X-100, and incubated with rabbit anti-BeGC1 antiserum. The reactivity was developed with a specific goat anti-rabbit IgG antibody conjugated with Alexa-Fluor 488 (Molecular Probes). The lipid droplets of the eyespot were visualized with the lipid-specific fluorescent dye Nile Red. From top left to bottom right, the following are shown: zoospore under phase contrast (differential interference contrast image), BeGC1 (green), lipid droplets (red) of the eyespot, and a merge of BeGC1 and lipid droplets images. The arrows indicate the position of the eyespot apparatus, and the arrowhead shows the zoospore flagellum. The images shown are at 1000× magnification.

We investigated the subcellular localization of the BeGC1 protein by raising antiserum against a recombinant polypeptide corresponding to the GC domain of BeGC1. As a control, we used antiserum against a cytoplasmic membrane-bound ATPase from *Blastocladiella* [32]. First, to investigate localization, we conducted western blot analysis on total zoospore extracts prepared under different centrifugation conditions. The BeGC1 antiserum recognized a single band of approximately 68 kDa, consistent with the predicted size of BeGC1. This band was detected only in the 12,000 × *g* pellet fraction, whereas the ATPase was detected in both the 12,000 × *g* pellet and the 100,000 × *g* pellet, indicating that part of the cytoplasmic membrane is also present in the 12,000 × *g* pellet (Figure 4A). This result suggests that BeGC1 is probably localized to a specific organelle recovered in the 12,000 × *g* fraction. Interestingly, the zoospore flagellar axoneme is also found in the 12,000 × *g* pellet [33]. This observation was confirmed by investigation of the presence of α-tubulin in the subcellular fractions analyzed, as this protein together with β-tubulin are major components of zoospore flagellum (Figure 4A) [33].

For further examination of the subcellular localization of BeGC1, immunofluorescence microscopy experiments were carried out. The data showed that BeGC1 is localized to a discrete site in the zoospores, in a position consistent with the eyespot, within the plasma membrane region, near the lipid granules identified using the dye Nile Red (Figure 4B) [4, 5].

### A Putative Cyclic Nucleotide Gated Channel Tied to Phototaxis

Using the genome data, we also identified a putative cyclic nucleotide-gated channel named BeCNG1. BeCNG1 shows similarity to the human rod photoreceptor cGMP-gated channel subunit alpha-1 (33.3% similarity) [34] and to the K^+^-selective cGMP-gated ion channel (31.6% similarity to the third repeat module of the channel [35]) that controls the chemosensation of sea urchin sperm [9]. Comparison of the putative pore helix region and the cGMP-binding site of BeCNG1 with other channels reveals the conservation of important amino acid residues (Figure S4). The K^+^ selectivity signature GYGD is present in BeCNG1 (Figure S4A), suggesting that it may act as a K^+^-selective channel. Furthermore, zoospores treated with the CNG inhibitor L-*cis*-diltiazem [36] were observed to stop swimming, suggesting a possible role of BeCNG1 in the control of flagellar beating. Investigation of expression levels of *BeCNG1* transcript during *Blastocladiella* sporulation (Figure S4C) revealed the same pattern observed for *BeGC1* transcript, consistent with its involvement in zoospore phototaxis.

### Nonstandard Route of GC Activaton in *B. emersonii*

The domain structure of BeGC1 is unprecedented, bringing together a type I rhodopsin sensory domain and a GC catalytic domain, suggesting that light directly triggers the synthesis of cGMP. The proposed mechanism of vertebrate ROS-GC activation is distinct, with no outside signal acting to directly stimulate GC activity (Figure 1A). However, the possibility that rhodopsin light stimulation acts as the external signal to directly activate ROS-GC has recently been suggested [37], with the proposed model bearing strong similarity to the mechanism described for BeGC1 activation (Figure 1B). Studies of the phototransduction cascade of scallop ciliary photoreceptors have also indicated the involvement of a putative membrane GC activated by light, with a light stimulus inducing an increase in cGMP and the consequent opening of light-dependent K^+^-selective channels [38]. Thus, the activation of GCs by light signal via rhodopsin stimulation may not be restricted to Blastocladiomycota fungi.

The present report shows that zoospores of the fungus *Blastocladiella emersonii* are capable of phototaxis toward green light, the selectivity for light of this particular wavelength being confirmed by zoospores depleated of carotenoids and with retinal complementation. These results are consistent with the involvement of rhodopsin in phototaxis. These data also reveal that the rhodopsin-photoreceptor constitutes the N-terminal domain of a novel guanylyl cyclase enzyme in which an S helix motif connects the rhodopsin domain to the guanylyl cyclase domain. Such protein module most likely transmits the light signal from the rhodopsin domain to the GC domain in BeGC1 [23]. The immunolocalization of BeGC1 to the eyespot apparatus of zoospores is consistent with the proposed role of this organelle as a photoreceptive structure. Additionally, the finding of a putative cGMP-gated channel encoded in *Blastocladiella* genome suggests BeCNG1 as a likely component of the phototactic signaling cascade. Taken together, our data indicate that *Blastocladiella* builds visual perception structures with many similarities to component parts of vertebrate vision, with cGMP and rhodopsin acting in both signaling pathways (Figures 1A and 1B). The finding that both rhodopsin and guanylyl cyclase domains are encoded as a single protein with the light signal directly activating cGMP synthesis reveals a unique solution to the task of converting light perception into a cellular signal.

## Author Contributions

G.M.A. performed all experimental work. P.A.Z. constructed the microfluidic chamber and helped with the phototaxis experiments. R.I.S. supervised the immunofluorescence experiments. G.L. and T.A.R. performed genome assembly and analyses and gene phylogenies. G.M.A., T.A.R., and S.L.G. wrote the manuscript and participated in detailed discussion of study design and data analysis at all stages of the study. S.L.G. designed and supervised the project.

## Accession Numbers

The GenBank accession numbers for the genes reported in this paper are as follows: *BeGC1*, KF309499; *BeCNG1*, KF309500; *bifunctional lycopene cyclase/phytoene synthase*, KJ468785; *phytoene dehydrogenase*, KJ468786; and *carotenoid dioxygenase*, KJ468787.

## Supplemental Information

Document S1. Figures S1–S4 and Supplemental Experimental Procedures

Table S1. List of Genomes Used for Comparative Analysis and Phylogenetic Analysis, Related to Figures S2 and S3 and the Supplemental Experimental Procedures

Document S2. Article plus Supplemental Information
