# Supplementary material for: A Rhodopsin-Guanylyl Cyclase Gene Fusion Functions in Visual Perception in a Fungus
Source: Curr Biol. 2014 Jun 2;24(11):1234–40. doi: 10.1016/j.cub.2014.04.009 (PMC4046227; doi:10.1016/j.cub.2014.04.009)
Supplement: Document S1. Figures S1–S4 and Supplemental Experimental Procedures [file mmc1.pdf]

Current Biology, Volume *25*

Supplemental Information

A Rhodopsin-Guanylyl Cyclase Gene Fusion Functions in Visual Perception in a Fungus

Gabriela M. Avelar, Robert I. Schumacher, Paulo A. Zaini, Guy Leonard, Thomas A. Richards, and Suely L. Gomes

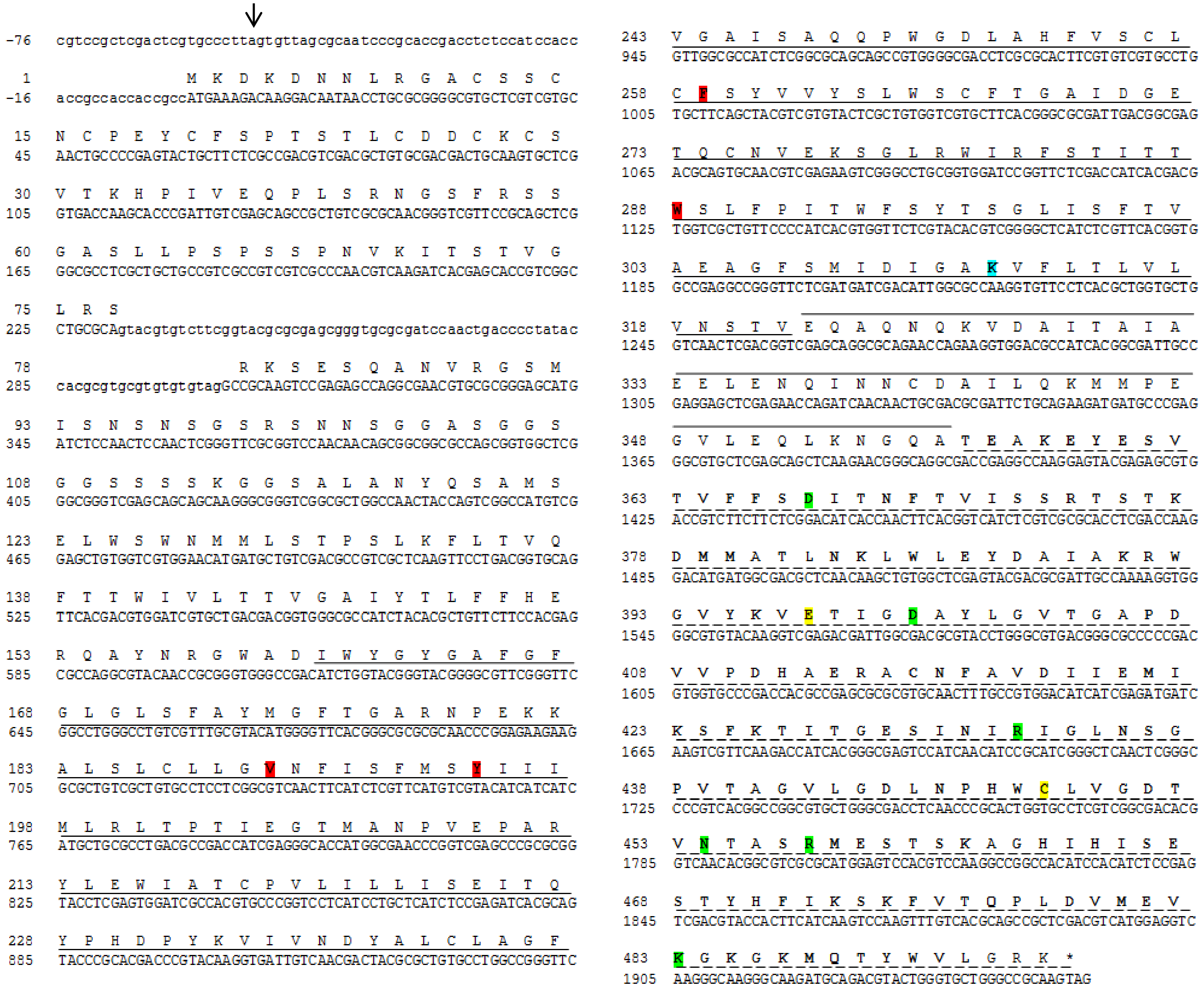

Figure S1. Nucleotide and deduced amino acid sequence of BeGC1 gene, Related to Figure 2. The arrow indicates the transcription start site determined in the 5´RACE assay. The rhodopsin domain (continuous line) and the guanylyl cyclase domain (dashed line) are underlined. The coiled-coil domain is overlined. In the Type I rhodopsin domain residues shown in red are conserved and make contact with retinal; the lysine that binds covalently to retinal (K255) is shown in cyan. In the guanylyl cyclase domain residues shown in green are important for catalysis and those in yellow recognize the guanine base in GTP.

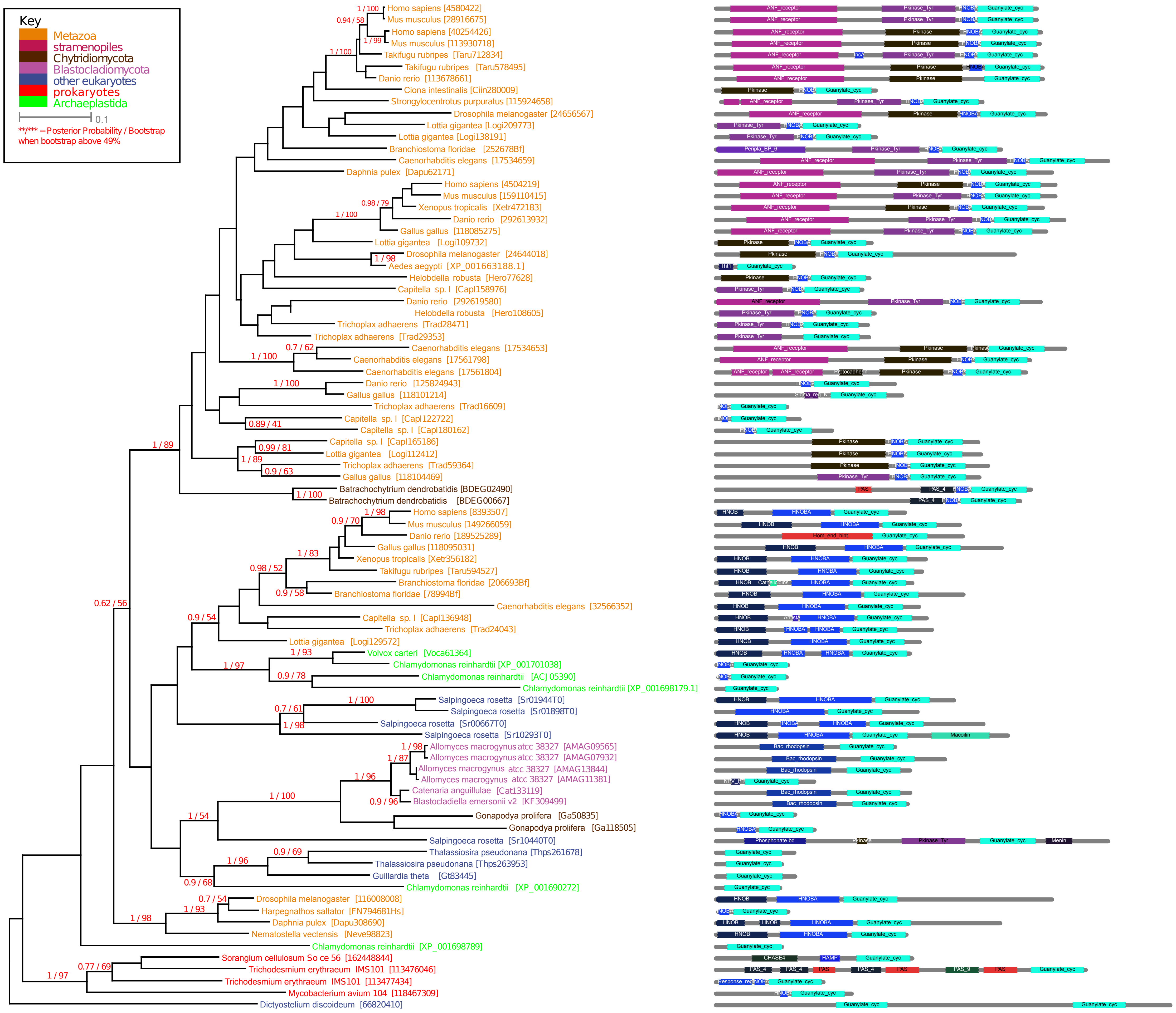

Key
Metazoa
stramenopiles
Chytridiomycota
Blastocladiomycota
other eukaryotes
prokaryotes
Archaeplastida
0.1
/* = Posterior Probability / Bootstrap when bootstrap above 49%
Homo sapiens [4580422]
Mus musculus [28916675]
Homo sapiens [40254426]
Mus musculus [113930718]
Takifugu rubripes [Taru712834]
Takifugu rubripes [Taru578495]
Danio rerio [113678661]
Ciona intestinalis [Ciin280009]
Strongylocentrotus purpuratus [115924658]
Drosophila melanogaster [24656567]
Lottia gigantea [Logi209773]
Lottia gigantea [Logi138191]
Branchiostoma floridae [252678Bf]
Caenorhabditis elegans [17534659]
Daphnia pulex [Dapu62171]
Homo sapiens [4504219]
Mus musculus [159110415]
Xenopus tropicalis [Xetr472183]
Danio rerio [292613932]
Gallus gallus [118085275]
Lottia gigantea [Logi109732]
Drosophila melanogaster [24644018]
Aedes aegypti [XP_001663188.1]
Helobdella robusta [Hero77628]
Capitella sp. I [Capl158976]
Danio rerio [292619580]
Helobdella robusta [Hero108605]
Trichoplax adhaerens [Trad28471]
Trichoplax adhaerens [Trad29353]
Caenorhabditis elegans [17534653]
Caenorhabditis elegans [17561798]
Caenorhabditis elegans [17561804]
Danio rerio [125824943]
Gallus gallus [118101214]
Trichoplax adhaerens [Trad16609]
Capitella sp. I [Capl122722]
Capitella sp. I [Capl180162]
Capitella sp. I [Capl165186]
Lottia gigantea [Logi112412]
Trichoplax adhaerens [Trad59364]
Gallus gallus [118104469]
Batrachochytrium dendrobatidis [BDEG02490]
Batrachochytrium dendrobatidis [BDEG00667]
Homo sapiens [8393507]
Mus musculus [149266059]
Danio rerio [189525289]
Gallus gallus [118095031]
Xenopus tropicalis [Xetr356182]
Takifugu rubripes [Taru594527]
Branchiostoma floridae [206693Bf]
Branchiostoma floridae [78994Bf]
Caenorhabditis elegans [32566352]
Capitella sp. I [Capl136948]
Trichoplax adhaerens [Trad24043]
Lottia gigantea [Logi129572]
Volvox carteri [Voca61364]
Chlamydomonas reinhardtii [XP_001701038]
Chlamydomonas reinhardtii [ACJ 05390]
Chlamydomonas reinhardtii [XP_001698179.1]
Salpingoeca rosetta [Sr01944T0]
Salpingoeca rosetta [Sr01898T0]
Salpingoeca rosetta [Sr00667T0]
Salpingoeca rosetta [Sr10293T0]
Allomyces macrogynus atcc 38327 [AMAG09565]
Allomyces macrogynus atcc 38327 [AMAG07932]
Allomyces macrogynus atcc 38327 [AMAG13844]
Allomyces macrogynus atcc 38327 [AMAG11381]
Catenaria anguillulae [Cat133119]
Blastocladiella emersonii v2 [KF309499]
Gonapodya prolifera [Ga50835]
Gonapodya prolifera [Ga118505]
Salpingoeca rosetta [Sr10440T0]
Thalassiosira pseudonana [Thps261678]
Thalassiosira pseudonana [Thps263953]
Guillardia theta [Gt83445]
Chlamydomonas reinhardtii [XP_001690272]
Drosophila melanogaster [116008008]
Harpegnathos saltator [FN794681Hs]
Daphnia pulex [Dapu308690]
Nematostella vectensis [Neve98823]
Chlamydomonas reinhardtii [XP_001698789]
Sorangium cellulosum So ce 56 [162448844]
Trichodesmium erythraeum IMS101 [113476046]
Trichodesmium erythraeum IMS101 [113477434]
Mycobacterium avium 104 [118467309]
Dictyostelium discoideum [66820410]
1 / 100
0.94 / 58
1 / 99
1 / 100
0.98 / 79
1 / 100
1 / 98
0.7 / 62
1 / 100
1 / 100
0.89 / 41
0.99 / 81
1 / 89
0.9 / 63
1 / 89
1 / 100
1 / 98
0.9 / 70
1 / 83
0.98 / 52
0.9 / 58
0.62 / 56
0.9 / 54
1 / 93
1 / 97
0.9 / 78
1 / 100
0.7 / 61
1 / 98
1 / 98
1 / 87
1 / 96
1 / 100
0.9 / 96
1 / 54
0.9 / 69
1 / 96
0.9 / 68
0.7 / 54
1 / 93
1 / 98
0.77 / 69
1 / 97

Figure S2. Phylogeny of the guanylyl-cyclase domain indicating a gene-fusion in the Blastocladiomycota fungi with a Type I rhodopsin domain, Related to Figure 2. Topology shown is a MrBayes phylogeny (100,000,000 generations, WAG+Γ, and a burn-in of 3,172 samples [sampled every 1,000 generations]). When consistent with the MrBayes topology and 50% or more bootstrap support values are added (from analysis in RAxML (LG + Γ) with 1,000 bootstraps). Support values in red: Bayesian posterior probability/ML bootstrap. Each branch is annotated with the PFAM domain architecture of the source sequence. The gene phylogeny includes a wider sampling of gene paralogues recovered from the sample dataset (Table S1) demonstrating cases of differential loss (for example in the 'chytrid' fungi sampled) and alternative protein domain architectures across this gene family as identified using PFAM [S1] All sequences are labelled with GenBank Accession numbers or genome sequencing centre Gene IDs. Sequences used in the alignment are available at https://github.com/guyleonard/blastocladiella/tree/master/publication/supplementary.

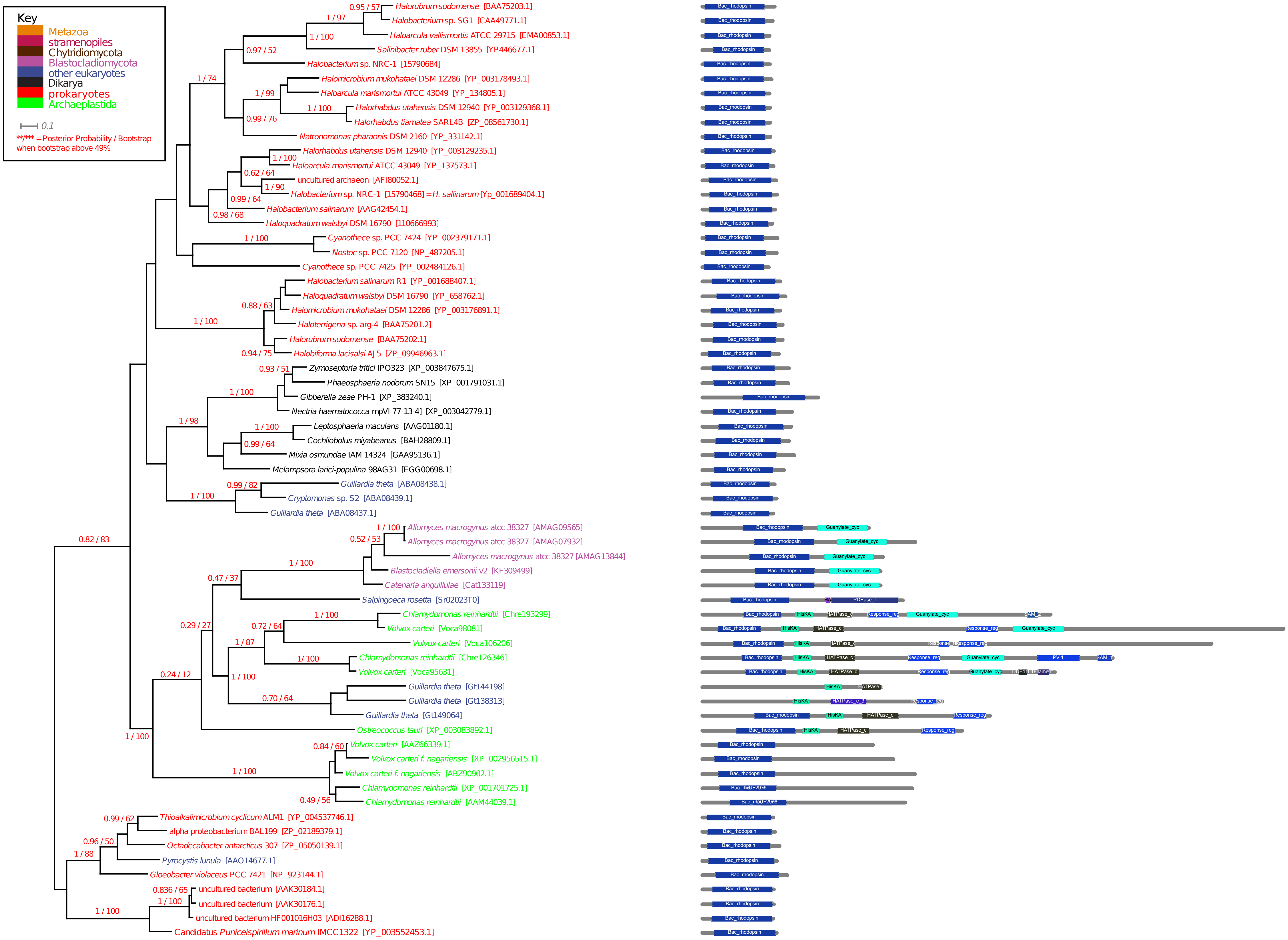
Key
Metazoa
stramenopiles
Chytridiomycota
Blastocladiomycota
other eukaryotes
Dikarya
prokaryotes
Archaeplastida
0.1
/* =Posterior Probability / Bootstrap when bootstrap above 49%
0.95 / 57 Halorubrum sodomense [BAA75203.1]
1 / 97 Halobacterium sp. SG1 [CAA49771.1]
1 / 100 Haloarcula vallismortis ATCC 29715 [EMA00853.1]
Salinibacter ruber DSM 13855 [YP446677.1]
0.97 / 52 Halobacterium sp. NRC-1 [15790684]
1 / 74
Halomicrobium mukohataei DSM 12286 [YP_003178493.1]
1 / 99 Haloarcula marismortui ATCC 43049 [YP_134805.1]
1 / 100 Halorhabdus utahensis DSM 12940 [YP_003129368.1]
Halorhabdus tiamatea SARL4B [ZP_08561730.1]
0.99 / 76 Natronomonas pharaonis DSM 2160 [YP_331142.1]
Halorhabdus utahensis DSM 12940 [YP_003129235.1]
1 / 100 Haloarcula marismortui ATCC 43049 [YP_137573.1]
0.62 / 64 uncultured archaeon [AFI80052.1]
1 / 90 Halobacterium sp. NRC-1 [15790468] =H. sallinarum [Yp_001689404.1]
0.99 / 64 Halobacterium salinarum [AAG42454.1]
0.98 / 68 Haloquadratum walsbyi DSM 16790 [110666993]
1 / 100 Cyanothece sp. PCC 7424 [YP_002379171.1]
Nostoc sp. PCC 7120 [NP_487205.1]
Cyanothece sp. PCC 7425 [YP_002484126.1]
Halobacterium salinarum R1 [YP_001688407.1]
Haloquadratum walsbyi DSM 16790 [YP_658762.1]
0.88 / 63 Halomicrobium mukohataei DSM 12286 [YP_003176891.1]
1 / 100 Haloterrigena sp. arg-4 [BAA75201.2]
Halorubrum sodomense [BAA75202.1]
0.94 / 75 Halobiforma lacisalsi AJ 5 [ZP_09946963.1]
0.93 / 51 Zymoseptoria tritici IPO323 [XP_003847675.1]
Phaeosphaeria nodorum SN15 [XP_001791031.1]
1 / 100 Gibberella zeae PH-1 [XP_383240.1]
Nectria haematococca mpVI 77-13-4] [XP_003042779.1]
1 / 98
1 / 100 Leptosphaeria maculans [AAG01180.1]
Cochliobolus miyabeanus [BAH28809.1]
0.99 / 64 Mixia osmundae IAM 14324 [GAA95136.1]
Melampsora larici-populina 98AG31 [EGG00698.1]
0.99 / 82 Guillardia theta [ABA08438.1]
1 / 100 Cryptomonas sp. S2 [ABA08439.1]
Guillardia theta [ABA08437.1]
0.82 / 83
1 / 100 Allomyces macrogynus atcc 38327 [AMAG09565]
0.52 / 53 Allomyces macrogynus atcc 38327 [AMAG07932]
Allomyces macrogynus atcc 38327 [AMAG13844]
1 / 100 Blastocladiella emersonii v2 [KF309499]
Catenaria anguillulae [Cat133119]
0.47 / 37 Salpingoeca rosetta [Sr02023T0]
1 / 100 Chlamydomonas reinhardtii [Chre193299]
0.29 / 27 0.72 / 64 Volvox carteri [Voca98081]
1 / 87 Volvox carteri [Voca106206]
1 / 100 Chlamydomonas reinhardtii [Chre126346]
0.24 / 12 Volvox carteri [Voca95631]
1 / 100 Guillardia theta [Gt144198]
0.70 / 64 Guillardia theta [Gt138313]
Guillardia theta [Gt149064]
Ostreococcus tauri [XP_003083892.1]
1 / 100
0.84 / 60 Volvox carteri [AAZ66339.1]
Volvox carteri f. nagariensis [XP_002956515.1]
1 / 100 Volvox carteri f. nagariensis [ABZ90902.1]
Chlamydomonas reinhardtii [XP_001701725.1]
0.49 / 56 Chlamydomonas reinhardtii [AAM44039.1]
0.99 / 62 Thioalkalimicrobium cyclicum ALM1 [YP_004537746.1]
alpha proteobacterium BAL199 [ZP_02189379.1]
0.96 / 50 Octadecabacter antarcticus 307 [ZP_05050139.1]
1 / 88 Pyrocystis lunula [AAO14677.1]
Gloeobacter violaceus PCC 7421 [NP_923144.1]
0.836 / 65 uncultured bacterium [AAK30184.1]
1 / 100 uncultured bacterium [AAK30176.1]
1 / 100 uncultured bacterium HF001016H03 [ADI16288.1]
Candidatus Puniceispirillum marinum IMCC1322 [YP_003552453.1]

Figure S3. Phylogeny of the putative Type I rhodopsin domain indicating a gene-fusion in the Blastocladiomycota fungi with a guanylyl-cyclase domain, Related to Figure 2. Topology shown is derived from a MrBayes analysis (100,000,000 generations, calculated using a WAG+Γ substitution matrix, and a burn-in of 5,957 samples [sampled every 1,000 generations]). When consistent with the MrBayes topology and 50% or more bootstrap support values are added. Bootstrap analysis was conducted using RAxML (LG+Γ substitution matrix) with 1,000 bootstraps). Support values are in red: Bayesian posterior probability/ML bootstrap. Each branch is annotated with the PFAM domain architecture of the source sequence. This analysis also showed a unique rhodopsin gene fusion architecture in the green algae that involves a histidine kinase, a response regulator and a putative adenylyl/guanylyl-cyclase domain. Based on the fusion architecture and the results of this phylogeny and the Blastocladiomycota guanylyl-cyclase domain phylogeny (Figure S2), we suggest that this is a convergent fusion. All sequences are labelled with GenBank Accession numbers or genome sequencing centre Gene IDs. Sequences used in the alignment are available at https://github.com/guyleonard/blastocladiella/tree/master/publication/supplementary.

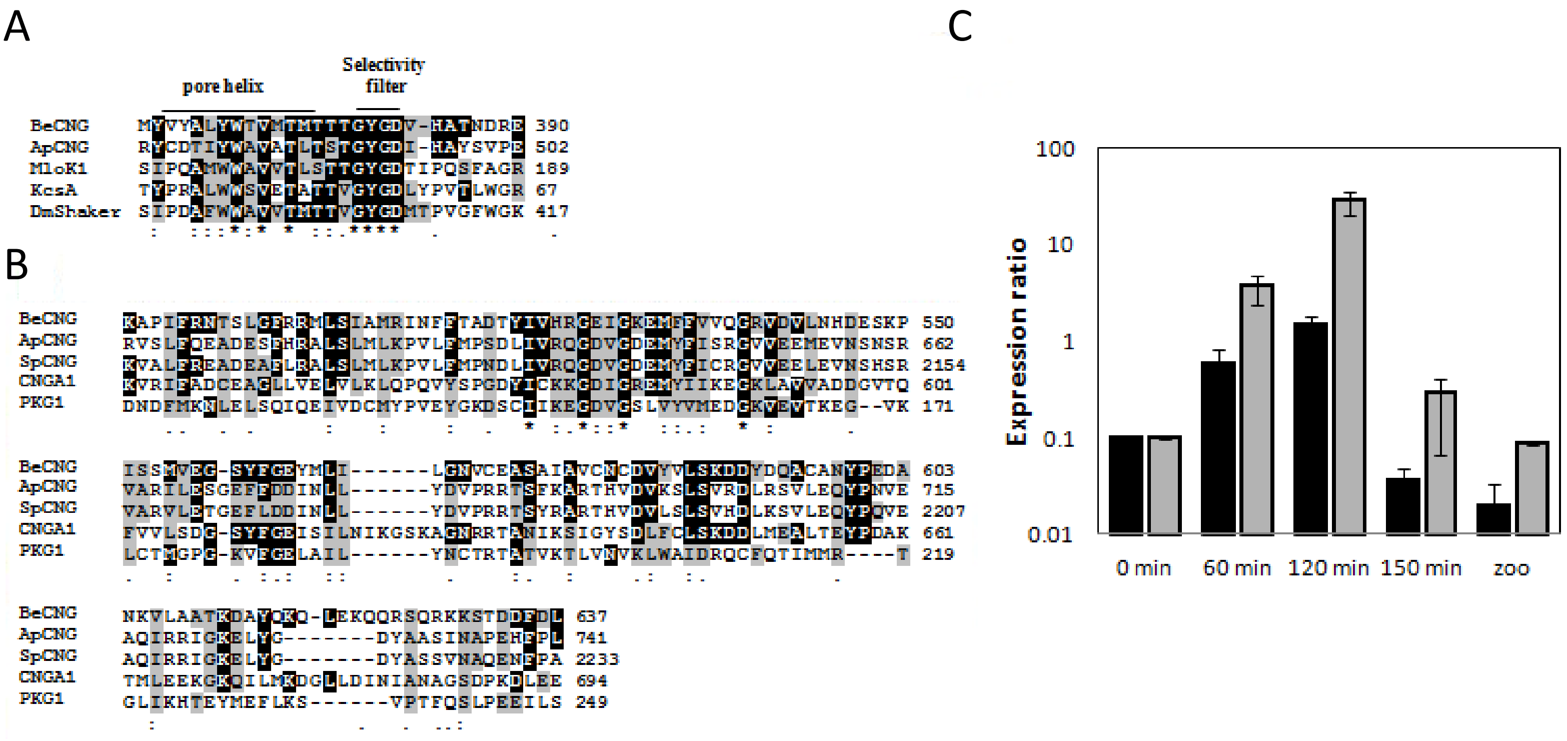

Figure S4 - Putative cyclic nucleotide gated channel BeCNG1: comparison of conserved sequence motifs (A and B) and transcript levels during *B. emersonii* sporulation, Related to Figure 1. (C). The sequences of the putative pore helix and selection filter are shown in **(A);** the cyclic nucleotide-binding domain is shown in **(B)**. The alignments include BeCNG1 (NCBI accession: KF309500); ApCNG, the cNMP gated potassium channel *Arbacia punctulata* (NCBI accession: ABH10136.1), MloK1, K+-selective CNG channel from *Mesorhizobium loti* (NCBI accession: NP_104392.1), KcsA, K+ channel from *Streptomyces lividans* (NCBI accession: Z37969.1), DmShaker, K+ channel from Drosophila (NCBI accession: X07131.1); SpCNG K+ channel from *Strongylocentrotus purpuratus* (NCBI accession: NM_001081964.1); CNGA1, cGMP-gated cation channel alpha-1 isoform 1 from *Homo sapiens* (NCBI accession: NP_001136036.1); PKG1, cGMP-binding site of PRKG1 (NCBI accession: NP_776703.1). Identical residues are marked with an asterisk (*), high conservation residues are marked with colons (:) and lower conservation residues are marked with a dot (.). The BeCNG channel was taken as reference. Residues identical to the reference are shown in black and conserved residues are shown in grey. **(C)** Transcript levels of *B. emersonii* BeCNG1 gene (black rectangles) evaluated by real-time RT-PCR at different times after induction of sporulation (0, 60, 120 and 150 min) and in the resulting zoospores (zoo). As a control, BeGC1 transcript levels (grey rectangles) were also analyzed. Data are mean values from three independent biological samples. Vertical bars represent standard deviation.

Supplemental Experimental Procedures

Genome sequencing of *Blastocladiella emersonii*.

Total DNA of a *Blastocladiella emersonii* isolate (ATCC 22665) was extracted from $4x10^9$ zoospores, as previously described [S2]. DNA was checked for both prokaryotic and eukaryotic contamination using general 18S and 16S SSU rDNA PCR. Each 50 µL PCR reactions contained 2 µL of each primer (10 $pM\mu L^{-1}$), 25 µL of Master Mix (Promega, containing 3 mM $MgCl_2$, 400 µM of each dNTP, and 50 U/mL of Taq DNA polymerase), 19 µL of PCR water, and 2 µL of a 1/1000 dilution of template DNA. Universal eukaryotic SSU rRNA primers 1F (5'CTGGTTGATCCTGCCAG-3') and 1520R (5'-CTGCAGGTTCACCTA-3') with the following cycling conditions were used; initial denaturation at 95 °C for 5 min, followed by 30 cycles of 95 °C for 1 min, 57 °C for 1 min, and 72 °C for 1.5 min, with a final extension at 72 °C for 10 min [S3]. For universal prokaryotic SSU rRNA amplification primers PA (5'-AGAGTTTGATCCTGGCTCAG-3') and PH (5'-AAGGAGGTCATCCAGCCGCA-3') [S4] with the following cycling conditions used, with *Escherichia coli* DNA acting as a positive control; initial denaturation of 94 °C for 5 min, followed by 30 cycles of 94 °C for 1 min, 55 °C for 1 min, 72 °C for 2 min, with a final step of 72 °C for 10 min.

The prokaryotic PCR was negative while the eukaryotic SSU PCR resulted in a single band of appropriate size. The 1F-1520R PCR reaction was purified using the Wizard ® SV gel and PCR clean-up system (Promega) and sequenced directly on both strands by Cogenics (Essex). Chromatograms were checked for inconsistencies, which could be the result of a multi-template amplification and the derived sequences used for BLASTn

(GenBank nr database) demonstrating the DNA was derived from a pure culture of the target microbe.

We sequenced the *Blastocladiella* genome using two approaches: 1) Two 454 FLX titanium (Roche) paired-end libraries, one at 3kb and the other at 20kb, and 2) Illumina GA2 paired-end 76nt sequencing. The DNA was prepared for both sequencing platforms using the standard protocols. The 454 approach generated 1,099,679 sequence reads resulting in 345,422,838 bp of sequences for the 3kb library and 189,318 sequence reads resulting in 67,116,270 bp of sequences for the 20kb library. Two lanes of Illumina GA2 paired-end 76bp sequencing was performed yielding a total of 41,083,984 reads. A total of 3,535,030,995 bp from all sequencing technologies. Quality filtering for the Illumina reads was achieved by removing bases 1-10 and 62-76.

Several assembly approaches were tested but finally the *Blastocladiella* genome sequence was assembled using a hybrid approach similar to that of the Fire Ant genome [S5]. Firstly the Velvet assembler (1.0.09) [S6] was used with the Illumina data and resulted in 37,387 contigs spanning 25,868,447bp at a mean coverage of 52x. Kmer size of 33 was used along with expected kmer coverage of 15 and a coverage cutoff of 0.9 as determined by the Velvet Optimiser 2.14 script bundled with Velvet. This produced an N50 contig-length of 2,594 bp. The EMBOSS tool 'splitter' was used to split up the Illumina-based contigs into pseudo-reads that were 400bp long with an overlap of 200p. The pseudo-reads were then used with the Roche 454 assembler 'newbler' (http://www.454.com/products/analysis-software/) also known as 'GS De Novo Assembler' version 2.5 with standard settings, along with the three 'sff' – flow gram – files from the 454 sequencing (total of 423,386,588 bp). This resulted in 14,099 contigs with an N50 of

3,912bp and an L50 of 52. Newbler also produced 772 scaffolds with an N50 of 155,559 bp. The estimated genome size is 60.4Mb and our total coverage estimate is 58.5x. CEGMA analysis [S7] demonstrated 19 partial genes missing (7 of which are missing from all three publically available Blastocladiomycota genome assemblies) from a list of 248 ‘core’ genes suggesting the genome assembly contains 95% of the predicted proteome.

Open reading frames for predicted proteins were identified using the MAKER pipeline [S8], which combined several gene-calling methods (MAKER, SNAP (http://korflab.ucdavis.edu/software.html) and Augustus [S9] and the *Blastocladiella* ESTs [S10] as a reference dataset (which had been manually checked to remove potential sources of contamination). Several iterations of the pipeline were carried out, similarly to the “MAKER Tutorial 2012” (http://gmod.org/wiki/MAKER_Tutorial_2012) help pages. Augustus was run using the reference genomes of *Rhizopus oryzae*, *Neurospora crassa*, *Ustilago maydis* and *Saccharomyces cerevisiae*. The output from these runs, the SNAP runs and MAKER were combined, de-duplicated, and clustered using the program CD-HIT with a 0.95% identity. The longest ORF for each cluster was picked as the representative of that cluster, resulting in 23,790 predicted ORFs for *Blastocladiella emersonii*. Each ORF was then BLAST searched locally against a database of genomes (see Table S1). All contigs that did not return a top hit that was not an opisthokont were manually inspected for evidence that they were contaminating sequences. These searches did not identify contaminant sequence reads.

Gene phylogenies

Each of the three individual domains derived from two genes (BeGC1: guanylyl cyclase enzyme domain and rhodopsin-like domain [GenBank Accession no. KF309499] and

BeCNG: putative K^+-selective channel [GenBank Accession no. KF309500) were used as seed sequence for a custom bioinformatic pipeline for generating phylogenies [S11], which uses BLASTp to recover a set of amino acid sequences (gathering threshold set to 1e-10) from a local database of published and publicly available genome databases. (Table S1). The retrieved sequences are then aligned using MAFFT v7.03b [S12] and then masked using the program TrimAl [S13] (to remove gaps and highly variant sites) and a preliminary tree calculated using FastTree [S14] with aLRT topology support values. These trees and alignments are used as guides for manual improved taxon sampling, sequence alignment, and alignment masking. Alignments were confined to sequences that we could achieve a reasonable gene alignment, in many cases minimizing sampling of the wider gene family (e.g. rhodopsin). For each dataset, long branches and closely related sequences from the same genus groups were removed and additional taxa sampled (using manual BLAST searching of NCBI nr database, Broad genome database and JGI Genome Portal). Once the set of sequences were finalised they were realigned with MAFFT and manually masked. These alignments were then passed to the program ProtTest3 [S15] to predict the 'best-fit' model of sequence substitution for phylogenetic analysis (details of the model are given in the figure legends that accompany each phylogeny). The settings predicted by ProtTest are then used - where possible - with the programs RAxML [S16] to generate a ML tree, (100 BKL and 1,000 BS) under the CAT model and with MrBayes3.2 (until the logLikelihood reaches a plateau for a minimum of 500,000 generation samples (sampled every 1,000 generations) – burnin calculated using Tracer v1.5 [S17]. Downstream scripts were used to produce annotated phylogenies with PFAM domains [1], multiple bootstrap/posterior probabilities and taxonomic classifications.

Phototaxis assay in growth media agar plates

Vegetative cells growing on PYG agar plates (0.13% peptone, 0.13% yeast extract, 0.3% glucose, and 1.5% agar) in the dark for 16h at 19°C were induced to sporulate also in the dark, by adding 2 ml of sterile water. The released zoospores were collected, filtered through nitex cloth, diluted in sterile water at a density of 2x10^{4}/ml. After maintaining the zoospores for 30 min in the dark they were inoculated in PYG agar plates to which 1 ml of sterile distilled water was previously added, to permit the zoospores to swim. Zoospores (50 µl) were carefully deposited on the plate in a position diametrically opposite to where the light beam was to be applied. The plates were immediately inserted individually inside black plastic envelopes. In the envelope that contained the plate to be exposed to the light beam, a hole of 0.5 cm in diameter was made in the plastic envelope in a position diametrically opposite to the point where the zoospores were inoculated. In the control plates no hole was made in the plastic envelope. Home-built LED light sources of 522 ± 17 nm wavelength and 4.4 mW/cm^2 intensity or 633 ± 13 nm wavelength and 4.4 mW/cm^2 intensity were applied over the plates, which were incubated at room temperature for 4h to allow the zoospores to germinate and enter vegetative growth. After further incubation at 17°C overnight, the plates were observed under a light microscope. The number of cells in the region of the light beam were counted and photographed. In the control plates, the corresponding region was also photographed and the number of cells counted. Phototaxis assays were also carried out after photobleaching of rhodopsin, by pre-incubating zoospores with hydroxylamine (500 µM) and green light (522 ± 17 nm wavelength; 4.4 mW/cm^2 intensity) for 10 min, and then repeating the procedure described above.

Phototaxis in the microfluidic chamber

Zoospores ($2x10^4$/ml) obtained as described in phototaxis in agar plates were kept in the dark for 30 min and then injected in an opaque microfluidic chamber containing a transparent area at the opposite end relative to the injection channel. Cells were observed with an inverted light microscope in a dark room to reduce environmental noise. Chamber dimensions were 100 µm in height, 1mm wide and 4cm long [S18]. The initial cell number was recorded at the transparent zone and cells were allowed to swim freely for 10 minutes, when cells were once again counted. The microscope light, which was used at three different wavelengths: green light (565 ± 25 nm; 55 $\mu W/cm^2$); blue light (465 ± 25 nm; 67 $\mu W/cm^2$) or red light (620 ± 30 nm; 35 $\mu W/cm^2$), was kept on during the 10 minutes as the "light" condition and kept off for the "dark" condition. The experiments with retinal analogs were carried out with zoospores obtained growing the fungus in the presence of the carotenogenesis inhibitor norflurazon (50 µM) for three generations, and then incubating the zoospores in the dark for 40 min with 5 µM of either retinalA1 or retinalA2 prior to phototaxis. Four independent biological replicas were performed for each condition and cell counts were normalized according to cell density. Statistical significance was assessed by a Student's T test with p-value cutoff of 0.05. Phototaxis experiments were also carried out in the presence of 10 µM of the GC inhibitor LY83583 [S19].

Determination of intracellular levels of cGMP

Zoospores obtained as described in phototaxis assays were collected, filtered through nitex cloth and diluted in sterile water at a density of $8x10^6$/ml. The suspension containing the zoospores was kept in the dark for at least 30 min before being exposed to a home-built

LED light source of wavelength of 522 ± 17 nm and 4.4 mW/cm^2 of intensity. After different times, aliquots of 0.9 ml were transferred to an eppendorf tube and used for determination of the amount of cGMP present in the sample. For that, the zoospores were lysed by adding 100 µl of 1M HCl and total cell extracts were immediately frozen in dry ice. The amount of cGMP was determined using the EIA Direct cyclic GMP kit (Sigma), which is a competitive immunoassay for the quantitative determination of cGMP. Changes in cGMP levels in zoospores exposed to green light were also determined after photobleaching of rhodopsin by pre-incubating zoospores with hydroxylamine (500 µM) and green light (522 ± 17 nm and 4.4 mW/cm^2 of intensity) for 10 min, followed by a 30 min-incubation in the dark. The zoospore suspension was then exposed to green light, and aliquots were collected at different time points for cGMP determination. All assays were made in biological triplicates. Levels of cGMP were also determined in experiments where endogenous retinal was substituted by retinalA1 using zoospores obtained from growth in the presence of the carotenogenesis inhibitor norflurazon (50 µM) for three generations, and then incubating or not with 5 µM of retinalA1 for 40 min in the dark prior to exposure to green light (522 ± 17 nm; 4.4 mW/cm^2) for different times.

Western Blot

Total zoospore extracts were prepared by freezing and thawing of the cells. Unbroken zoospores were separated by centrifugation of the cell lysate for 5 min at 1,000 x g. The supernatant was then fractionated by centrifugation of the cell extracts for 5 min at 12,000 x g. The resulting pellet (12,000 ppt) was saved, and the supernatant was further centrifuged at 100,000 x g for 10 min. All centrifugation steps were carried out at 4^oC. The resulting pellet (100,000 ppt) and supernatant (100,000 sup) were saved and all fractions were

analyzed by Western blotting. Equal amounts of protein from each fraction were separated by denaturing SDS-polyacrylamide gel electrophoresis, and the resolved proteins were transferred to nitrocellulose filter. Membranes were incubated overnight at 4°C with 1:1,000 dilution of anti-BeGC1 in Tris-HCl, pH 8.0, containing 150 mM NaCl, 0.002% Tween 20, and 0.03% Triton X-100. The blots were developed using the secondary antibody CF680 Goat Anti-rabbit IgG (Uniscience) at a 1:10,000 dilution. The bound complex was detected using the Odyssey Infrared Imaging System (Li-Cor Biosciences; Lincoln, NE). The antiserum against BeGC1 was obtained from a rabbit immunized with a recombinant polypeptide expressed in *E. coli* corresponding to the GC catalytic domain of BeGC1 fused to a histidine-tag at the N-terminus. As a control, we used an antiserum against BePAT1, a cytoplasmic membrane bound ATPase from *B. emersonii* [S20], and anti-α-tubulin (Sigma).

Immunofluorescence microscopy

Zoospores were collected by centrifugation at 1000 x g for 5 min and fixed with 4% p-formaldehyde, 1% calcium chloride for 30 min. After, the cells were permeabilized with PBS containing 0.1% Triton X-100, blocked with 1% BSA and incubated at 37°C for 30 min with a rabbit anti-BeGC1 antiserum (1:50) The reactivity was developed with specific Goat anti-rabbit IgG antibodies conjugated to Alexa-Fluor 488 (Molecular Probes). For the visualization of the lipid droplets of the eyespot, a lipid-specific fluorescent dye Nile Red (Invitrogen) was added to the zoospore suspension at a dilution of 1:10,000. Images taken in a Nikon Eclipse E600 microscope were obtained under identical settings and were submitted to deconvolution with Huygens Essential image processing program.

Rapid Amplification of cDNA Ends (5'RACE)

Rapid amplification of the 5' cDNA end (5'RACE) was performed according to instructions from the GeneRacer™ RACE Ready cDNA Kit (Invitrogen), using the primers included in the kit and gene specific primers (GSPs) designed based on expressed sequence tags (GSP1GC1: 5´CGAGAACCGGATCCACCGCAGGCCCG3´ and GSP2GC1: 5´ATGAAGTTGACGCCGAGGAGGCACAGC3´) [S10]. RACE products from the PCR were cloned in pGEM®-T vector system (Promega) and sequenced.

Real-time reverse transcription-PCR (qRT-PCR)

Five micrograms of total RNA were reverse transcribed using 200 U of SuperScriptIII reverse transcriptase (Invitrogen) and 500 ng of random nonamers according to manufacturer's instructions. Approximately 180 ng of the resulting cDNA were used as template in the PCR assay, which included 800 nM of the forward and reverse primers and 10 µl of Platinum SYBR Green qPCR SuperMix UDG (Applied Biosystems). Primers used were as follows: BeGC′1, 5 -GACATCTGGTACGGGTACGG-3′ (forward) and 5′-GGAGATGAGCAGGATGAGGA-3′ (reverse), BeCNG, 5′-GAATTCCCTGACGTGGTGGGACTACT-3′ (forward) and 5′-GCGGCCGCTCTCGAACAGGTCAAAGTCG-3′ (reverse). Quantitative real time RT-PCR experiments (qRT-PCR) were performed using the GeneAmp 5700 Sequence Detection System (Applied Biosystems) equipment and the thermocycling conditions comprised an initial step at 50 °C for 2 min, followed by 95 °C for 10 min, 40 cycles of 95 °C for 15 s, and 60 °C for 1 min. For each gene and sporulation point analyzed, three independent RNA samples were used. The gene encoding the mitochondrial RNA helicase-

like protein was used as the calibrator gene in all experiments. Determination of the expression ratios was carried out using the $\mathbf{2^{-\Delta\Delta CT}}$ method [S21].

S1. Bateman, A., Coin, L., Durbin, R., Finn, R.D., Hollich, V., Griffiths-Jones, S., Khanna, A., Marshall, M., Moxon, S., Sonnhammer, E.L., et al. (2004). The Pfam protein families database. Nucleic Acids Res *32*, D138-141.

S2. Marques Mdo, V., and Gomes, S.L. (1992). Cloning and structural analysis of the gene for the regulatory subunit of cAMP-dependent protein kinase in *Blastocladiella emersonii*. J Biol Chem *267*, 17201-17207.

S3. Lefevre, E., Bardot, C., Noel, C., Carrias, J.F., Viscogliosi, E., Amblard, C., and Sime-Ngando, T. (2007). Unveiling fungal zooflagellates as members of freshwater picoeukaryotes: evidence from a molecular diversity study in a deep meromictic lake. Environ Microbiol *9*, 61-71.

S4. Edwards, U., Rogall, T., Blocker, H., Emde, M., and Bottger, E.C. (1989). Isolation and direct complete nucleotide determination of entire genes. Characterization of a gene coding for 16S ribosomal RNA. Nucleic Acids Res *17*, 7843-7853.

S5. Wurm, Y., Wang, J., Riba-Grognuz, O., Corona, M., Nygaard, S., Hunt, B.G., Ingram, K.K., Falquet, L., Nipitwattanaphon, M., Gotzek, D., et al. (2011). The genome of the fire ant Solenopsis invicta. Proc Natl Acad Sci U S A *108*, 5679-5684.

S6. Zerbino, D.R., and Birney, E. (2008). Velvet: algorithms for de novo short read assembly using de Bruijn graphs. Genome Res *18*, 821-829.

S7. Parra, G., Bradnam, K., Ning, Z., Keane, T., and Korf, I. (2009). Assessing the gene space in draft genomes. Nucleic Acids Res *37*, 289-297.

S8. Cantarel, B.L., Korf, I., Robb, S.M., Parra, G., Ross, E., Moore, B., Holt, C., Sanchez Alvarado, A., and Yandell, M. (2008). MAKER: an easy-to-use annotation pipeline designed for emerging model organism genomes. Genome Res *18*, 188-196.

S9. Stanke, M., and Morgenstern, B. (2005). AUGUSTUS: a web server for gene prediction in eukaryotes that allows user-defined constraints. Nucleic Acids Res *33*, W465-467.

S10. Ribichich, K.F., Salem-Izacc, S.M., Georg, R.C., Vencio, R.Z., Navarro, L.D., and Gomes, S.L. (2005). Gene discovery and expression profile analysis through sequencing of expressed sequence tags from different developmental stages of the chytridiomycete *Blastocladiella emersonii*. Eukaryot Cell *4*, 455-464.

S11. Richards, T.A., Soanes, D.M., Foster, P.G., Leonard, G., Thornton, C.R., and Talbot, N.J. (2009). Phylogenomic analysis demonstrates a pattern of rare and ancient horizontal gene transfer between plants and fungi. Plant Cell *21*, 1897-1911.

S12. Katoh, K., and Standley, D.M. (2013). MAFFT multiple sequence alignment software version 7: improvements in performance and usability. Mol Biol Evol *30*, 772-780.

S13. Capella-Gutierrez, S., Silla-Martinez, J.M., and Gabaldon, T. (2009). trimAl: a tool for automated alignment trimming in large-scale phylogenetic analyses. Bioinformatics *25*, 1972-1973.

S14. Price, M.N., Dehal, P.S., and Arkin, A.P. (2010). FastTree 2--approximately maximum-likelihood trees for large alignments. PLoS One *5*, e9490.
S15. Darriba, D., Taboada, G.L., Doallo, R., and Posada, D. (2011). ProtTest 3: fast selection of best-fit models of protein evolution. Bioinformatics *27*, 1164-1165.
S16. Stamatakis, A. (2006). RAxML-VI-HPC: maximum likelihood-based phylogenetic analyses with thousands of taxa and mixed models. Bioinformatics *22*, 2688-2690.
S17. Rambaut, A.D., A.J. (2007). Tracer v1.5 [computer program]. Avaliable: http://beast.bio.ed.ac.uk/Tracer.
S18. Meng, Q.H., Zhou, L.X., Luo, J.L., Cao, J.P., Tong, J., and Fan, S.J. (2005). Effect of 7-hydroxystaurosporine on glioblastoma cell invasion and migration. Acta Pharmacol Sin *26*, 492-499.
S19. Schmidt, M.J., Sawyer, B.D., Truex, L.L., Marshall, W.S., and Fleisch, J.H. (1985). LY83583: an agent that lowers intracellular levels of cyclic guanosine 3',5'-monophosphate. J Pharmacol Exp Ther *232*, 764-769.
S20. Fietto, L.G., Pugliese, L., and Gomes, S.L. (2002). Characterization and expression of two genes encoding isoforms of a putative Na, K-ATPase in the chytridiomycete *Blastocladiella emersonii*. Biochim Biophys Acta *1576*, 59-69.
S21. Livak, K.J., and Schmittgen, T.D. (2001). Analysis of relative gene expression data using real-time quantitative PCR and the 2(-Delta Delta C(T)) Method. Methods *25*, 402-408.
