# Supplementary material for: A Rhodopsin-Guanylyl Cyclase Gene Fusion Functions in Visual Perception in a Fungus
Source: Curr Biol. 2014 Jun 2;24(11):1234–40. doi: 10.1016/j.cub.2014.04.009 (PMC4046227; doi:10.1016/j.cub.2014.04.009)
Supplement: Document S2. Article plus Supplemental Information [file mmc3.pdf]

Current Biology 24, 1234–1240, June 2, 2014 ©2014 The Authors http://dx.doi.org/10.1016/j.cub.2014.04.009

Report

A Rhodopsin-Guanylyl Cyclase Gene Fusion Functions in Visual Perception in a Fungus

Gabriela M. Avelar,[1] Robert I. Schumacher,[1] Paulo A. Zaini,[1] Guy Leonard,[2] Thomas A. Richards,[2,*] and Suely L. Gomes[1,*]
[1]Departamento de Bioquímica, Instituto de Química, Universidade de São Paulo, São Paulo 05508-000, Brazil
[2]Biosciences, University of Exeter, Geoffrey Pope Building, Stocker Road, Exeter EX4 4QD, UK

Summary

Sensing light is the fundamental property of visual systems, with vision in animals being based almost exclusively on opsin photopigments [1]. Rhodopsin also acts as a photoreceptor linked to phototaxis in green algae [2, 3] and has been implicated by chemical means as a light sensor in the flagellated swimming zoospores of the fungus *Allomyces reticulatus* [4]; however, the signaling mechanism in these fungi remains unknown. Here we use a combination of genome sequencing and molecular inhibition experiments with light-sensing phenotype studies to examine the signaling pathway involved in visual perception in the closely related fungus *Blastocladiella emersonii.* Our data show that in these fungi, light perception is accomplished by the function of a novel gene fusion (*BeGC1*) of a type I (microbial) rhodopsin domain and guanylyl cyclase catalytic domain. Photobleaching of rhodopsin function prevents accumulation of cGMP levels and phototaxis of fungal zoospores exposed to green light, whereas inhibition of guanylyl cyclase activity negatively affects fungal phototaxis. Immunofluorescence microscopy localizes the BeGC1 protein to the external surface of the zoospore eyespot positioned close to the base of the swimming flagellum [4, 5], demonstrating this is a photoreceptive organelle composed of lipid droplets. Taken together, these data indicate that Blastocladiomycota fungi have a cGMP signaling pathway involved in phototaxis similar to the vertebrate vision-signaling cascade but composed of protein domain components arranged as a novel gene fusion architecture and of distant evolutionary ancestry to type II rhodopsins of animals.

Results and Discussion

Cyclic GMP is an important signaling molecule controlling a large spectrum of physiological responses in eukaryotes. In vertebrates, for example, this system functions with photoreceptors in visual perception (Figure 1A) [6–8]. It is unclear what aspects of this visual perception system are present in other eukaryotes. The fungus *Allomyces reticulatus* forms swimming zoospores and has been suggested to use rhodopsin-mediated signaling to initiate phototaxis [4]. Nevertheless, how the light signal is transmitted to direct flagellar beating is unknown. Publicly available fungal genomes, mostly from ascomycetes and basidiomycetes (Dikarya), demonstrate that these fungi encode opsins, phytochromes, and cryptochromes [10]. However, all of these fungi lack a motile life cycle stage powered by a flagellum, and therefore phototaxis is not observed. *Blastocladiella emersonii* is a close relative of *Allomyces*, branching below the Dikarya and Glomeromycota fungi [11]. Nearly four decades ago, it was demonstrated that the presence of cGMP along with guanylyl cyclase and cGMP phosphodiesterase activities correlated with the completion of *Blastocladiella* sporulation stage during biogenesis of flagellated zoospores [12–14]. These reports indicate the presence of a cGMP signaling pathway, an observation supported by the identification of cDNAs encoding putative guanylyl cyclases and a cGMP phosphodiesterase in *Blastocladiella* transcriptome [15, 16]. In contrast the cGMP pathway appears to be absent in all Dikarya fungi [17].

Genome Sequencing Data Reveal a Novel Guanylyl Cyclase in *B. emersonii*

To identify *Blastocladiella* cGMP signaling pathway, we sequenced the genome using second-generation sequencing methods. The genome data allowed us to identify the complete nucleotide sequence of a novel guanylyl cyclase-encoding gene (*BeGC1*) with a unique protein domain architecture containing a C-terminal GC catalytic domain and an N-terminal rhodopsin domain, representing a novel gene fusion (Figure S1 available online). We confirmed that this gene architecture is transcribed as a single gene using 5′ rapid amplification of cDNA ends (RACE) data (Figure S1), while quantitative RT-PCR (qRT-PCR) indicates that *BeGC1* transcript is highly expressed in late sporulation cells, during zoospore biogenesis [16]. The *BeGC1* gene is the only rhodopsin found in the draft assembly of *Blastocladiella* genome, with only one melanopsin also being present in the assembled genome. Melanopsin is a photosensitive protein involved in regulating circadian rhythms and other nonvisual reponses to light, with maximum sensitivities near to 480 nm (blue light) [18]. Additional searches of the Blastocladiomycota *Allomyces macrogynus* and *Catenaria anguillulae* genome assemblies confirmed that these fungi also possess the rhodopsin-guanylyl cyclase gene fusion, but with four recent duplications of the *BeGC1* ortholog in *Allomyces* genome and a fission and/or loss of the type I rhodopsin domain in one of these duplication forms (Figure S2); the phenomenon of domain loss and/or fission has been observed frequently in fungi [19]. Orthologs of the *BeGC1* gene were unidentified in all other fungal genome data sets searched (Table S1), including flagellated fungi (checked June 2013).

The predicted BeGC1 amino acid sequence (626 residues, calculated 68 kDa) demonstrated that residues that putatively interact with the chromophore retinal are conserved, including the lysine known to form a Shiff base (Figure S1). Interestingly, the *Blastocladiella* genome contains the genes necessary for carotenoid biosynthesis (bifunctional lycopene cyclase/phytoene synthase, phytoene dehydrogenase, and carotenoid dioxygenase). Analysis of the C-terminal portion of BeGC1 showed high similarity (65%) to the GC domain of a retinal guanylyl cyclase from *Aedes aegypt* with amino acids that

*Correspondence: t.a.richards@exeter.ac.uk (T.A.R.), sulgomes@iq.usp.br (S.L.G.)
This is an open access article under the CC BY license (http://creativecommons.org/licenses/by/3.0/).

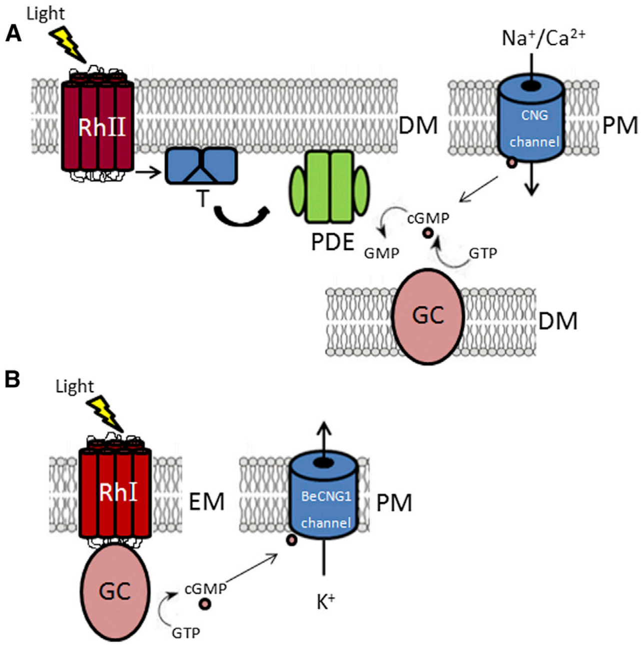

Figure 1. Schematic Models of the Signaling Pathway of Vertebrate Rod Photoreceptor and *Blastocladiella* Zoospore Phototaxis

(A) In the vertebrate visual signaling pathway, photoexcitation of the G protein-coupled rhodopsin receptor leads to activation of rod and cone heterotrimeric G protein transducin complex stimulating hydrolysis of cGMP [6]. The decrease in cGMP concentration leads to closure of cGMP-gated (CNG) channels, blockage of Na^+ influx, and hyperpolarization of photoreceptor plasma membrane, leading to transmission of signal through synapses [6]. Reduction of CNG channel activity blocks Ca^{2+} influx, decreasing cytoplasmic calcium concentration in retinal photoreceptor outer segments (ROS) and leading to activation of guanylyl cyclases (ROS-GCs) by the now Ca^{2+}-free GC-activating proteins (GCAPs) through acceleration of ROS-GC dimerization, thus restoring cGMP levels [7, 8].

(B) In the *B. emersonii* zoospore phototaxis transduction pathway, photoisomerization of rhodopsin in BeGC1 activates guanylyl cyclase activity, leading to the synthesis of cGMP from GTP. Cyclic GMP opens K^+-selective BeCNG1 channels, thereby causing hyperpolarization of the plasma membrane. A putative opening of voltage-activated calcium channels could produce elevation of $[Ca^{2+}]$, which would interact with the flagellum altering the flagellar beat, as in *Arbacia* sperm [9].

RhI, type I rhodopsin; RhII, type II rhodopsin; GC, guanylyl cyclase; T, transducin; PDE, phosphodiesterase; EM, eyespot membrane; PM, plasma membrane; DM, disk membrane. See also Figure S4.

distinguish guanylyl from adenylyl cyclases present (Figure S1). Using homology-based 3D structure modeling, we identified a third protein module: a coiled-coil (CC) domain linking the rhodopsin and GC domains, encompassing 47 amino acids, and with high structural similarity to the CC domain found in all mammalian guanylyl cyclases (Figure 2), a feature shown to be important in regulation and signaling of mammalian guanylyl cyclases [21, 23]. Its function is to prevent constitutive activation of GCs and transmit the activating signal to the catalytic domain [21, 24].

Phylogenetic Analysis of BeGC1 Guanylyl Cyclase and Rhodopsin Domains

To investigate the evolutionary ancestry of the BeGC1 protein, we conducted phylogenetic analysis of the component domains. The guanylyl cyclase domain phylogeny demonstrates that the Blastocladiomycota gene fusion branches with an unfused putative GC domain homolog from the Chytridiomycota *Gonapodya prolifera* with strong support (Figure S2). The rest of the tree includes a collection of eukaryotic algae, opisthokonts (e.g., animals and fungi), and a sequence from the Amoebozoan *Dictyostelium discoideum*. The patchy taxon distribution and low tree resolution make it difficult to identify the evolutionary ancestry of this domain.

Rhodopsins are seven-transmembrane α helix membrane proteins with a retinal cofactor and have been identified in prokaryotes and eukaryotes. Rhodopsins are classified into two groups, type I and type II, which are also named microbial and metazoan rhodopsins, respectively [25]. There is little sequence identity between type I and type II rhodopsins, making it difficult to align these genes for phylogenetic analysis, although ancestral state reconstruction and analysis of structural similarities have demonstrated that these two gene groups are distantly related [26, 27].

The rhodopsin phylogenetic analysis was restricted to a subsection of the gene family in order to improve tree resolution, demonstrating that Blastocladiomycota rhodopsins branch with type I rhodopsins. The phylogeny showed a very patchy taxon distribution, including prokaryotic sequences, environmental sequences, eukaryotic algae, the choanoflagellate *Salpingoeca rosetta*, and some Dikarya fungi. The Dikarya rhodopsin sequences branch separately from the BeGC1 cluster as a distant paralog (Figure S3). The phylogenetic resolution was poor with Blastocladiomycota sequences branching within a cluster composed of the protist *Salpingoeca* and eukaryotic algae, making it difficult to pinpoint the ancestry of the rhodopsin domain. However, these results demonstrate that the Blastocladiomycota type I rhodopsin-like domain is of distant evolutionary derivation to the type II rhodopsins of animal vision.

Rhodopsin-Guanylyl Cyclase Activity Is Essential for Phototaxis in *B. emersonii*

For identification of the function of BeGC1, *Blastocladiella* zoospores were exposed to a green light beam source (522 nm) similar to the wavelength at which *Allomyces* zoospores presented their peak (536 nm) phototaxis behavior [4]. *Blastocladiella* zoospores were inoculated in growth media agar plates in a position opposite the light beam. The fraction of zoospores germinated at the light source were counted and compared to the numbers obtained in control plates, which were not exposed to light. These experiments demonstrated a 5-fold increase in zoosporangia under the light source relative to control plates (Figures 3A and 3B).

As a reliable gene knockout protocol is not available for *Blastocladiella*, to test whether rhodopsin is involved in zoospores phototaxis, we used a photobleaching protocol [28] to suppress the rhodopsin function by pre-exposing zoospores to hydroxylamine and green light before performing phototaxis assays at a concentration that did not affect zoospore swimming capacity. A 4-fold reduction in zoosporangia colonization was observed within the light exposed region (Figure 3A).

To check the influence of light wavelength in phototaxis, we conducted the same experiment using a red light source (633 nm), which resulted in less than half the number of zoosporangia colonizing the illuminated region (Figure 3A), demonstrating a preference for green light consistent with results for *Allomyces* phototaxis [4].

To confirm green light phototaxis in *Blastocladiella*, we used direct microscope observations to investigate the movement of zoospores along a microfluidic glass chamber. Zoospores

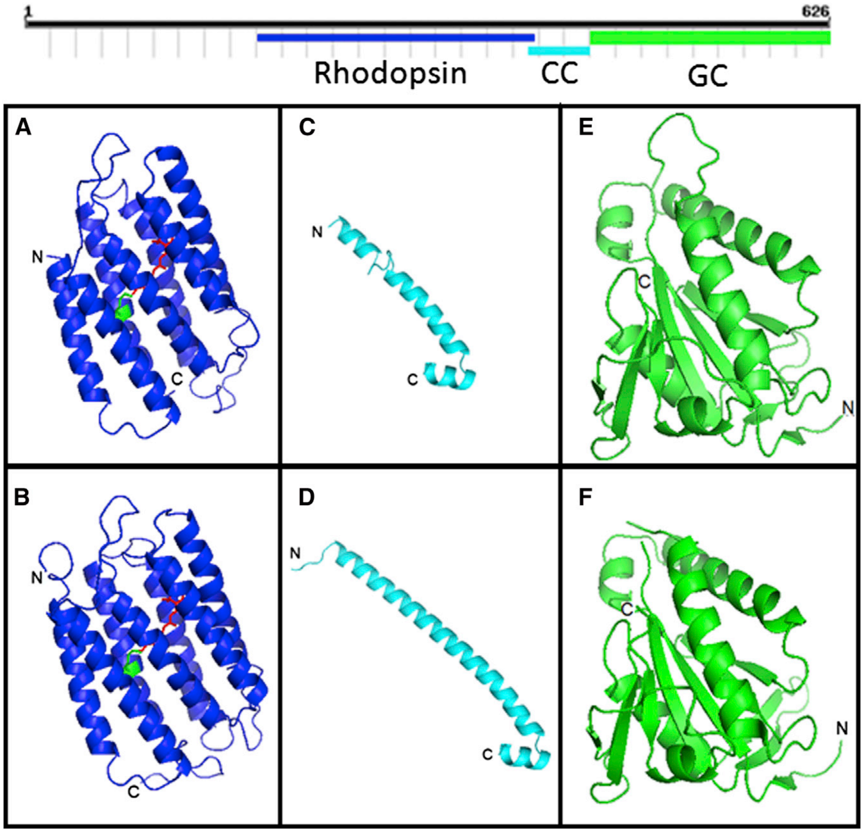

Figure 2. Structural Features of BeGC1 Protein Domains Constructed by Swiss-Model Homology-Based Approach

(A and B) The BeGC1 rhodopsin domain structure in (A) is based on the crystal structure of *Halobacterium salinarum* type I rhodopsin [20] shown in (B), with the retinal denoted in red and the lysine of the Shiff base in green.

(C and D) The structure of the coiled-coil domain, which links the rhodopsin domain to the GC domain on BeGC1, in (C) is based on the crystal structure of *Rattus norvegicus* soluble guanylyl cyclase CC domain [21] shown in (D).

(E and F) The structure of BeGC1 guanylyl cyclase catalytic domain in (E) is based on the crystal structure of the catalytic domain of soluble guanylyl cyclase CYG12 from *Chlamydomonas reinhardtii* [22] shown in (F).

See also Figures S1–S3.

were inoculated at one side of the chamber, and their accumulation was observed under a light microscope in the area opposite to the inoculum, illuminated with light of different wavelengths. Zoospores in this region of the chamber were counted before and after 10 min of illumination, and the numbers indicated that green light is about 2-fold more efficient at promoting phototaxis than is blue light or red light (Figure 3C).

To further establish the selectivity of zoospore phototaxis, we inhibited carotenogenis by growing *Blastocladiella* for three generations in the presence of the inhibitor norflurazon [29]. Treated zoospores demonstrated normal swimming but were incapable of performing phototaxis. However, after incubation with retinalA1, zoospore phototaxis was restored, with green light again being the preferential stimulus compared to red light (Figure 3D). In contrast, when zoospores were incubated with retinalA2, phototaxis was also restored, but red light was the preferential stimulus (Figure 3D), consistent with data demonstrating that retinalA2 serves as a chromophore in red-shifted visual pigments [30]. These data also demonstrate that the rhodopsin is acting as the primary light sensor in our experimental conditions as this shift from green light to red light sensitivity with the addition of retinalA2 is characteristic of rhodopsin rather than melanopsin function [30].

To investigate whether guanylyl cyclase activity is involved in zoospore phototaxis, we determined the intracellular cGMP levels in zoospores exposed to green light for different periods of time using a competitive immunoassay that permits the quantitative determination of cGMP. The levels of cGMP showed a rapid and short-lived increase when zoospores were exposed to green light (Figure 3E), indicating that GC activity in zoospores peaks by 5 s after green light exposure. To check whether rhodopsin function is linked to GC activation in response to green light, we repeated the experiment with zoospores that had undergone photobleaching [28]. In this experiment no increase in GC activity was coupled with green light exposure (Figure 3E), demonstrating that light activation of rhodopsin is linked to GC activation. The levels of cGMP were also investigated in phototaxis experiments with zoospores obtained from cells grown in the presence of norflurazon and incubated with retinalA1 or not incubated. In the presence of retinalA1 and green light, cGMP levels increased significantly upon irradiation, whereas without retinalA1 no increase was observed (Figure 3F). These results indicate that a retinylidene protein is necessary for triggering changes in cGMP levels during green light exposure. We also analyzed phototaxis in *Blastocladiella* zoospores incubated with the guanylyl cyclase inhibitor LY83583 [31] at a concentration at which no effect on completion of the life cycle or on zoospore swimming is observed. The number of zoospores present in the illuminated region was 3.5-fold lower in the presence of LY83583 than in its absence, consistent with GC activity in phototaxis (Figure 3D).

BeGC1 Is Localized to the Zoospore "Eyespot" Apparatus

We investigated the subcellular localization of the BeGC1 protein by raising antiserum against a recombinant polypeptide corresponding to the GC domain of BeGC1. As a control, we used antiserum against a cytoplasmic membrane-bound ATPase from *Blastocladiella* [32]. First, to investigate localization, we conducted western blot analysis on total zoospore extracts prepared under different centrifugation conditions. The BeGC1 antiserum recognized a single band of approximately 68 kDa, consistent with the predicted size of BeGC1. This band was detected only in the 12,000 × *g* pellet fraction, whereas the ATPase was detected in both the 12,000 × *g* pellet and the 100,000 × *g* pellet, indicating that part of the cytoplasmic membrane is also present in the 12,000 × *g* pellet (Figure 4A). This result suggests that BeGC1 is probably localized to a specific organelle recovered in the 12,000 × *g* fraction. Interestingly, the zoospore flagellar axoneme is also found in

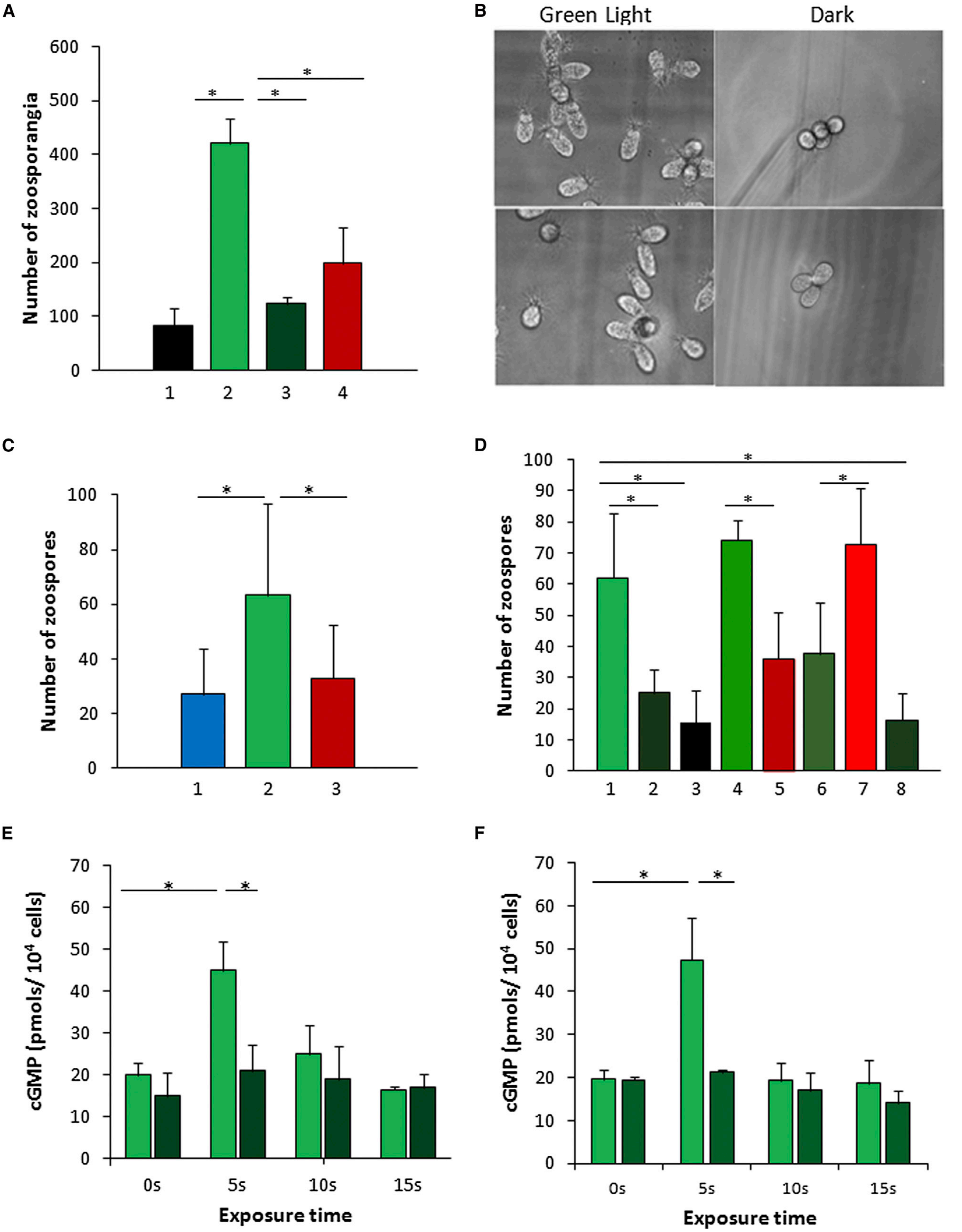

Figure 3. Phototaxis of *B. emersonii* Zoospores Involves Rhodopsin and Guanylyl Cyclase Activity

(A and B) Data of phototaxis assays in agar plates. The resulting vegetative cells found in the region of the plates exposed (column 2) or not (column 1) to green light (522 ± 17 nm; 4.4 mW/cm^2) and preincubated (column 3) or not (column 2) with 500 μM hydroxylamine (HA) were visualized under a light

(legend continued on next page)

the 12,000 × *g* pellet [33]. This observation was confirmed by investigation of the presence of α-tubulin in the subcellular fractions analyzed, as this protein together with β-tubulin are major components of zoospore flagellum (Figure 4A) [33].

For further examination of the subcellular localization of BeGC1, immunofluorescence microscopy experiments were carried out. The data showed that BeGC1 is localized to a discrete site in the zoospores, in a position consistent with the eyespot, within the plasma membrane region, near the lipid granules identified using the dye Nile Red (Figure 4B) [4, 5].

A Putative Cyclic Nucleotide Gated Channel Tied to Phototaxis

Using the genome data, we also identified a putative cyclic nucleotide-gated channel named BeCNG1. BeCNG1 shows similarity to the human rod photoreceptor cGMP-gated channel subunit alpha-1 (33.3% similarity) [34] and to the K^+-selective cGMP-gated ion channel (31.6% similarity to the third repeat module of the channel [35]) that controls the chemosensation of sea urchin sperm [9]. Comparison of the putative pore helix region and the cGMP-binding site of BeCNG1 with other channels reveals the conservation of important amino acid residues (Figure S4). The K^+ selectivity signature GYGD is present in BeCNG1 (Figure S4A), suggesting that it may act as a K^+-selective channel. Furthermore, zoospores treated with the CNG inhibitor L-*cis*-diltiazem [36] were observed to stop swimming, suggesting a possible role of BeCNG1 in the control of flagellar beating. Investigation of expression levels of *BeCNG1* transcript during *Blastocladiella* sporulation (Figure S4C) revealed the same pattern observed for *BeGC1* transcript, consistent with its involvement in zoospore phototaxis.

Nonstandard Route of GC Activaton in *B. emersonii*

The domain structure of BeGC1 is unprecedented, bringing together a type I rhodopsin sensory domain and a GC catalytic domain, suggesting that light directly triggers the synthesis of cGMP. The proposed mechanism of vertebrate ROS-GC activation is distinct, with no outside signal acting to directly stimulate GC activity (Figure 1A). However, the possibility that rhodopsin light stimulation acts as the external signal to directly activate ROS-GC has recently been suggested [37], with the proposed model bearing strong similarity to the mechanism described for BeGC1 activation (Figure 1B). Studies of the phototransduction cascade of scallop ciliary photoreceptors have also indicated the involvement of a putative membrane GC activated by light, with a light stimulus inducing an increase in cGMP and the consequent opening of light-dependent K^+-selective channels [38]. Thus, the activation of GCs by light signal via rhodopsin stimulation may not be restricted to Blastocladiomycota fungi.

The present report shows that zoospores of the fungus *Blastocladiella emersonii* are capable of phototaxis toward green light, the selectivity for light of this particular wavelength being confirmed by zoospores depleated of carotenoids and with retinal complementation. These results are consistent with the involvement of rhodopsin in phototaxis. These data also reveal that the rhodopsin-photoreceptor constitutes the N-terminal domain of a novel guanylyl cyclase enzyme in which an S helix motif connects the rhodopsin domain to the guanylyl cyclase domain. Such protein module most likely transmits the light signal from the rhodopsin domain to the GC domain in BeGC1 [23]. The immunolocalization of BeGC1 to the eyespot apparatus of zoospores is consistent with the proposed role of this organelle as a photoreceptive structure. Additionally, the finding of a putative cGMP-gated channel encoded in *Blastocladiella* genome suggests BeCNG1 as a likely component of the phototactic signaling cascade. Taken together, our data indicate that *Blastocladiella* builds visual perception structures with many similarities to component parts of vertebrate vision, with cGMP and rhodopsin acting in both signaling pathways (Figures 1A and 1B). The finding that both rhodopsin and guanylyl cyclase domains are encoded as a single protein with the light signal directly activating cGMP synthesis reveals a unique solution to the task of converting light perception into a cellular signal.

Accession Numbers

The GenBank accession numbers for the genes reported in this paper are as follows: *BeGC1*, KF309499; *BeCNG1*, KF309500; *bifunctional lycopene cyclase/phytoene synthase*, KJ468785; *phytoene dehydrogenase*, KJ468786; and *carotenoid dioxygenase*, KJ468787.

Supplemental Information

Supplemental Information includes four figures, Supplemental Experimental Procedures, and one table and can be found with this article online at http://dx.doi.org/10.1016/j.cub.2014.04.009.

Author Contributions

G.M.A. performed all experimental work. P.A.Z. constructed the microfluidic chamber and helped with the phototaxis experiments. R.I.S. supervised the immunofluorescence experiments. G.L. and T.A.R. performed genome assembly and analyses and gene phylogenies. G.M.A., T.A.R., and S.L.G. wrote the manuscript and participated in detailed discussion of study

microscope and cells were counted (A) and photographed (B). Vegetative cells were also counted in plates exposed to red light (633 ± 13 nm; 4.4 mW/cm^2; column 4). Results are mean values of three biological replicates.

(C) Phototaxis in microfluidic chamber. Data are from zoospores exposed to blue light (465 ± 25 nm; 67 μW/cm^2; column 1), zoospores exposed to green light (565 ± 25 nm; 55 μW/cm^2; column 2) and zoospores exposed to red light (620 ± 30 nm; 35 μW/cm^2; column 3).

(D) Phototaxis in microfluidic chamber with zoospores from growth with norflurazon. Zoospores from growth without (column 1) or with 10 μM (column 2) or 50 μM (column 3) norflurazon exposed to green light, zoospores from growth with 50 μM norflurazon reconstituted with 5 μM retinalA1 exposed to green light (565 ± 25 nm; 55 μW/cm^2; column 4) or red light (620 ± 30 nm; 35 μW/cm^2; column 5) or reconstituted with 5 μM retinalA2 exposed to green light (565 ± 25 nm; 55 μW/cm^2; column 6) or red light (620 ± 30 nm; 35 μW/cm^2; column 7), and zoospores from growth without norflurazon incubated with 10 μM of GC inhibitor LY83583 exposed to green light (565 ± 25 nm; 55 μW/cm^2; column 8) are shown. Results are mean values of three biological replicates.

(E) Changes in intracellular cGMP levels in zoospores upon green light irradiation. Levels of cGMP were determined before and after different times of zoospore exposure to green light (522 ± 17 nm; 4.4 mW/cm^2) and in the absence (green rectangles) or presence (dark green rectangles) of 500 μM hydroxylamine.

(F) Changes in cGMP levels after different times of irradiation with green light (522 ± 17 nm; 4.4 mW/cm^2) of zoospores obtained in the presence of 50 μM norflurazon and incubated (green rectangles) or not incubated (dark green rectangles) with 5 μM retinalA1 to restore phototactic capacity. Data are mean values of three independent replicates.

Error bars indicate the SE. Asterisks denote significant differences at $^*p < 0.05$.

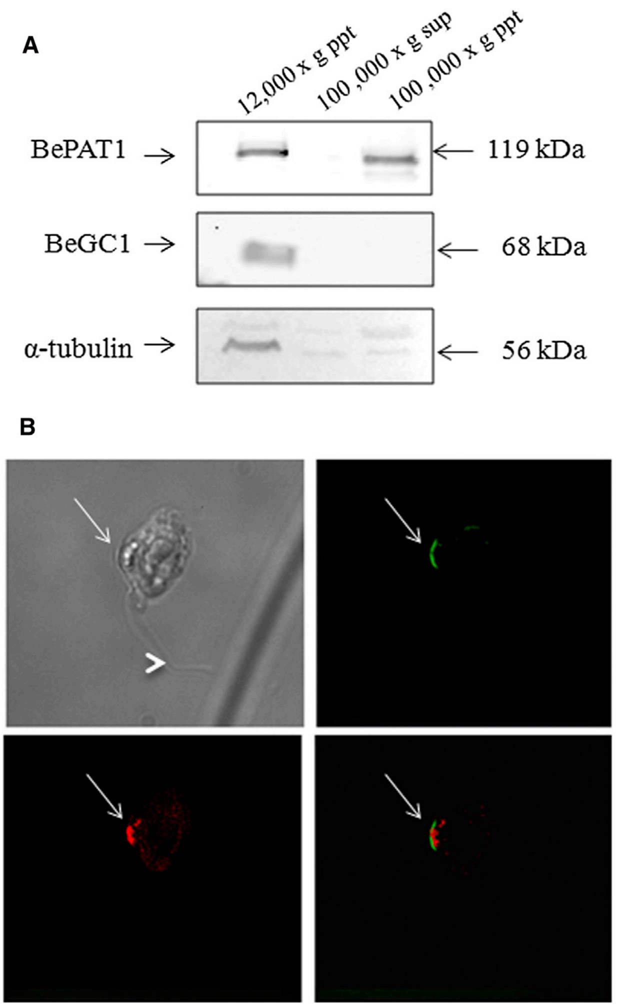

Figure 4. Subcellular Localization of BeGC1 Protein

(A) Western blot analysis of subcellular fractions of zoospore lysates obtained by differential centrifugation, as described in the Supplemental Experimental Procedures. Fractions were resolved through SDS-PAGE followed by western blotting and were developed using rabbit antisera against BeGC1, BePAT1, and α-tubulin, as well as the fluorescent CF680 Goat anti-rabbit IgG as a secondary antibody. The bound complexes were detected using the Odyssey Infrared Imaging System.

(B) Localization of BeGC1 by immunofluorescence microscopy. Zoospores were fixed with 4% p-formaldehyde and 1% calcium chloride, permeabilized with PBS containing 0.1% Triton X-100, and incubated with rabbit anti-BeGC1 antiserum. The reactivity was developed with a specific goat anti-rabbit IgG antibody conjugated with Alexa-Fluor 488 (Molecular Probes). The lipid droplets of the eyespot were visualized with the lipid-specific fluorescent dye Nile Red. From top left to bottom right, the following are shown: zoospore under phase contrast (differential interference contrast image), BeGC1 (green), lipid droplets (red) of the eyespot, and a merge of BeGC1 and lipid droplets images. The arrows indicate the position of the eyespot apparatus, and the arrowhead shows the zoospore flagellum. The images shown are at 1000× magnification.

design and data analysis at all stages of the study. S.L.G. designed and supervised the project.

Acknowledgments

The authors thank Mauricio Baptista for generously providing the LED light sources, Frederico Gueiros-Filho for the use of the fluorescence microscope, and Konrad Paszkiewicz for genome sequencing support. We also acknowledge the kind gift of retinalA2 from Rosalie Crouch and John Oatis.

This work was supported by a grant to S.L.G. from Fundação de Amparo à Pesquisa do Estado de São Paulo (FAPESP). T.A.R. is an EMBO Young Investigator, and his research group is supported by grants from the Moore Foundation, FP6 BIODIVERSA, Leverhulme, NERC, and BBSRC. G.L. is supported by BBSRC grant BB/G00885X/1. G.M.A. and P.Z. are, respectively, predoctoral and postdoctoral fellows of FAPESP, and S.L.G. is partially supported by Conselho Nacional de Desenvolvimento Científico e Tecnológico (CNPq). The funders had no role in study design, data collection and analysis, decision to publish, or preparation of the manuscript.

Received: September 10, 2013
Revised: March 3, 2014
Accepted: April 3, 2014
Published: May 15, 2014

References

1. Fain, G.L., Hardie, R., and Laughlin, S.B. (2010). Phototransduction and the evolution of photoreceptors. Curr. Biol. *20*, R114–R124.
2. Foster, K.W., Saranak, J., Patel, N., Zarilli, G., Okabe, M., Kline, T., and Nakanishi, K. (1984). A rhodopsin is the functional photoreceptor for phototaxis in the unicellular eukaryote *Chlamydomonas*. Nature *311*, 756–759.
3. Sineshchekov, O.A., Jung, K.H., and Spudich, J.L. (2002). Two rhodopsins mediate phototaxis to low- and high-intensity light in *Chlamydomonas reinhardtii*. Proc. Natl. Acad. Sci. USA *99*, 8689–8694.
4. Saranak, J., and Foster, K.W. (1997). Rhodopsin guides fungal phototaxis. Nature *387*, 465–466.
5. Cantino, E.C., and Truesdell, L.C. (1970). Organization and fine structure of the side body and its lipid sac in the zoospore of *Blastocladiella emersonii*. Mycologia *62*, 548–567.
6. Zhang, X., and Cote, R.H. (2005). cGMP signaling in vertebrate retinal photoreceptor cells. Front. Biosci. *10*, 1191–1204.
7. Frins, S., Bönigk, W., Müller, F., Kellner, R., and Koch, K.W. (1996). Functional characterization of a guanylyl cyclase-activating protein from vertebrate rods. Cloning, heterologous expression, and localization. J. Biol. Chem. *271*, 8022–8027.
8. Yu, H., Olshevskaya, E., Duda, T., Seno, K., Hayashi, F., Sharma, R.K., Dizhoor, A.M., and Yamazaki, A. (1999). Activation of retinal guanylyl cyclase-1 by Ca2+-binding proteins involves its dimerization. J. Biol. Chem. *274*, 15547–15555.
9. Strünker, T., Weyand, I., Bönigk, W., Van, Q., Loogen, A., Brown, J.E., Kashikar, N., Hagen, V., Krause, E., and Kaupp, U.B. (2006). A K+-selective cGMP-gated ion channel controls chemosensation of sperm. Nat. Cell Biol. *8*, 1149–1154.
10. Idnurm, A., Verma, S., and Corrochano, L.M. (2010). A glimpse into the basis of vision in the kingdom Mycota. Fungal Genet. Biol. *47*, 881–892.
11. James, T.Y., Letcher, P.M., Longcore, J.E., Mozley-Standridge, S.E., Porter, D., Powell, M.J., Griffith, G.W., and Vilgalys, R. (2006). A molecular phylogeny of the flagellated fungi (Chytridiomycota) and description of a new phylum (Blastocladiomycota). Mycologia *98*, 860–871.
12. Silverman, P.M. (1976). Regulation of guanylate cyclase activity during cytodifferentiation of *Blastocladiella emersonii*. Biochem. Biophys. Res. Commun. *70*, 381–388.
13. Silverman, P.M., and Epstein, P.M. (1975). Cyclic nucleotide metabolism coupled to cytodifferentiation of *Blastocladiella emersonii*. Proc. Natl. Acad. Sci. USA *72*, 442–446.
14. Vale, M.R., Gomes, S.L., and Maia, J.C. (1975). Independent cAMP and cGMP phosphodiesterases in *Blastocladiella emersonii*. FEBS Lett. *56*, 332–336.
15. Ribichich, K.F., Salem-Izacc, S.M., Georg, R.C., Vêncio, R.Z., Navarro, L.D., and Gomes, S.L. (2005). Gene discovery and expression profile analysis through sequencing of expressed sequence tags from different developmental stages of the chytridiomycete Blastocladiella emersonii. Eukaryot. Cell *4*, 455–464.
16. Vieira, A.L.G., Linares, E., Augusto, O., and Gomes, S.L. (2009). Evidence of a Ca(2+)-(*)NO-cGMP signaling pathway controlling zoospore biogenesis in the aquatic fungus *Blastocladiella emersonii*. Fungal Genet. Biol. *46*, 575–584.
17. Johnson, J.L., and Leroux, M.R. (2010). cAMP and cGMP signaling: sensory systems with prokaryotic roots adopted by eukaryotic cilia. Trends Cell Biol. *20*, 435–444.

18. Bailes, H.J., and Lucas, R.J. (2013). Human melanopsin forms a pigment maximally sensitive to blue light (λmax ≈ 479 nm) supporting activation of G(q/11) and G(i/o) signalling cascades. Proc. Biol. Sci. *280*, 20122987.
19. Leonard, G., and Richards, T.A. (2012). Genome-scale comparative analysis of gene fusions, gene fissions, and the fungal tree of life. Proc. Natl. Acad. Sci. USA *109*, 21402–21407.
20. Sass, H.J., Büldt, G., Gessenich, R., Hehn, D., Neff, D., Schlesinger, R., Berendzen, J., and Ormos, P. (2000). Structural alterations for proton translocation in the M state of wild-type bacteriorhodopsin. Nature *406*, 649–653.
21. Ma, X., Beuve, A., and van den Akker, F. (2010). Crystal structure of the signaling helix coiled-coil domain of the beta1 subunit of the soluble guanylyl cyclase. BMC Struct. Biol. *10*, 2.
22. Winger, J.A., Derbyshire, E.R., Lamers, M.H., Marletta, M.A., and Kuriyan, J. (2008). The crystal structure of the catalytic domain of a eukaryotic guanylate cyclase. BMC Struct. Biol. *8*, 42.
23. Saha, S., Biswas, K.H., Kondapalli, C., Isloor, N., and Visweswariah, S.S. (2009). The linker region in receptor guanylyl cyclases is a key regulatory module: mutational analysis of guanylyl cyclase C. J. Biol. Chem. *284*, 27135–27145.
24. Anantharaman, V., Balaji, S., and Aravind, L. (2006). The signaling helix: a common functional theme in diverse signaling proteins. Biol. Direct *1*, 25.
25. Spudich, J.L., Yang, C.S., Jung, K.H., and Spudich, E.N. (2000). Retinylidene proteins: structures and functions from archaea to humans. Annu. Rev. Cell Dev. Biol. *16*, 365–392.
26. Shen, L., Chen, C., Zheng, H., and Jin, L. (2013). The evolutionary relationship between microbial rhodopsins and metazoan rhodopsins. ScientificWorldJournal *2013*, 435651.
27. Devine, E.L., Oprian, D.D., and Theobald, D.L. (2013). Relocating the active-site lysine in rhodopsin and implications for evolution of retinylidene proteins. Proc. Natl. Acad. Sci. USA *110*, 13351–13355.
28. Hegemann, P., Hegemann, U., and Foster, K.W. (1988). Reversible bleaching of *Chlamydomonas reinhardtii* rhodopsin in vivo. Photochem. Photobiol. *48*, 123–128.
29. Boger, P., and Sandmann, G. (1983). Pigment biosyntheses and herbicides interaction. Photosynthetica *28*, 481–493.
30. Sineshchekov, O.A., Govorunova, E.G., Wang, J., and Spudich, J.L. (2012). Enhancement of the long-wavelength sensitivity of optogenetic microbial rhodopsins by 3,4-dehydroretinal. Biochemistry *51*, 4499–4506.
31. Schmidt, M.J., Sawyer, B.D., Truex, L.L., Marshall, W.S., and Fleisch, J.H. (1985). LY83583: an agent that lowers intracellular levels of cyclic guanosine 3′,5′-monophosphate. J. Pharmacol. Exp. Ther. *232*, 764–769.
32. Fietto, L.G., Pugliese, L., and Gomes, S.L. (2002). Characterization and expression of two genes encoding isoforms of a putative Na, K-ATPase in the chytridiomycete *Blastocladiella emersonii*. Biochim. Biophys. Acta *1576*, 59–69.
33. Abe, S.S., and Lovett, J.S. (1982). Microtubular proteins and tubulin pool changes during zoospore germination in the fungus *Blastocladiella emersonii*. Arch. Microbiol. *131*, 323–329.
34. Pittler, S.J., Lee, A.K., Altherr, M.R., Howard, T.A., Seldin, M.F., Hurwitz, R.L., Wasmuth, J.J., and Baehr, W. (1992). Primary structure and chromosomal localization of human and mouse rod photoreceptor cGMP-gated cation channel. J. Biol. Chem. *267*, 6257–6262.
35. Bönigk, W., Loogen, A., Seifert, R., Kashikar, N., Klemm, C., Krause, E., Hagen, V., Kremmer, E., Strünker, T., and Kaupp, U.B. (2009). An atypical CNG channel activated by a single cGMP molecule controls sperm chemotaxis. Sci. Signal. *2*, ra68.
36. Plachetzki, D.C., Fong, C.R., and Oakley, T.H. (2010). The evolution of phototransduction from an ancestral cyclic nucleotide gated pathway. Proc. Biol. Sci. *277*, 1963–1969.
37. Bondarenko, V.A., Hayashi, F., Usukura, J., and Yamazaki, A. (2010). Involvement of rhodopsin and ATP in the activation of membranous guanylate cyclase in retinal photoreceptor outer segments (ROS-GC) by GC-activating proteins (GCAPs): a new model for ROS-GC activation and its link to retinal diseases. Mol. Cell. Biochem. *334*, 125–139.
38. Gomez, M.P., and Nasi, E. (2000). Light transduction in invertebrate hyperpolarizing photoreceptors: possible involvement of a Go-regulated guanylate cyclase. J. Neurosci. *20*, 5254–5263.

Current Biology, Volume *25*

Supplemental Information

A Rhodopsin-Guanylyl Cyclase Gene Fusion Functions in Visual Perception in a Fungus

Gabriela M. Avelar, Robert I. Schumacher, Paulo A. Zaini, Guy Leonard, Thomas A. Richards, and Suely L. Gomes

```
        ↓
-76   cgtccgctcgactcgtgcccttagtgttagcgcaatcccgcaccgacctctccatccacc

1                     M  K  D  K  D  N  N  L  R  G  A  C  S  S  C
-16   accgccaccaccgccATGAAAGACAAGGACAATAACCTGCGCGGGGCGTGCTCGTCGTGC

15    N  C  P  E  Y  C  F  S  P  T  S  T  L  C  D  D  C  K  C  S
45    AACTGCCCCGAGTACTGCTTCTCGCCGACGTCGACGCTGTGCGACGACTGCAAGTGCTCG

30    V  T  K  H  P  I  V  E  Q  P  L  S  R  N  G  S  F  R  S  S
105   GTGACCAAGCACCCGATTGTCGAGCAGCCGCTGTCGCGCAACGGGTCGTTCCGCAGCTCG

60    G  A  S  L  L  P  S  P  S  S  P  N  V  K  I  T  S  T  V  G
165   GGCGCCTCGCTGCTGCCGTCGCCGTCGTCGCCCAACGTCAAGATCACGAGCACCGTCGGC

75    L  R  S
225   CTGCGCAgtacgtgtcttcggtacgcgcgagcgggtgcgcgatccaactgacccctatac

78                          R  K  S  E  S  Q  A  N  V  R  G  S  M
285   cacgcgtgcgtgtgtgtagGCCGCAAGTCCGAGAGCCAGGCGAACGTGCGCGGGAGCATG

93    I  S  N  S  N  S  G  S  R  S  N  N  S  G  G  A  S  G  G  S
345   ATCTCCAACTCCAACTCGGGTTCGCGGTCCAACAACAGCGGCGGCGCCAGCGGTGGCTCG

108   G  G  S  S  S  S  K  G  G  S  A  L  A  N  Y  Q  S  A  M  S
405   GGCGGGTCGAGCAGCAGCAAGGGCGGGTCGGCGCTGGCCAACTACCAGTCGGCCATGTCG

123   E  L  W  S  W  N  M  M  L  S  T  P  S  L  K  F  L  T  V  Q
465   GAGCTGTGGTCGTGGAACATGATGCTGTCGACGCCGTCGCTCAAGTTCCTGACGGTGCAG

138   F  T  T  W  I  V  L  T  T  V  G  A  I  Y  T  L  F  F  H  E
525   TTCACGACGTGGATCGTGCTGACGACGGTGGGCGCCATCTACACGCTGTTCTTCCACGAG

153   R  Q  A  Y  N  R  G  W  A  D  I  W  Y  G  Y  G  A  F  G  F
585   CGCCAGGCGTACAACCGCGGGTGGGCCGACATCTGGTACGGGTACGGGGCGTTCGGGTTC

168   G  L  G  L  S  F  A  Y  M  G  F  T  G  A  R  N  P  E  K  K
645   GGCCTGGGCCTGTCGTTTGCGTACATGGGGTTCACGGGCGCGCGCAACCCGGAGAAGAAG

183   A  L  S  L  C  L  L  G  V  N  F  I  S  F  M  S  Y  I  I  I
705   GCGCTGTCGCTGTGCCTCCTCGGCGTCAACTTCATCTCGTTCATGTCGTACATCATCATC

198   M  L  R  L  T  P  T  I  E  G  T  M  A  N  P  V  E  P  A  R
765   ATGCTGCGCCTGACGCCGACCATCGAGGGCACCATGGCGAACCCGGTCGAGCCCGCGCGG

213   Y  L  E  W  I  A  T  C  P  V  L  I  L  L  I  S  E  I  T  Q
825   TACCTCGAGTGGATCGCCACGTGCCCGGTCCTCATCCTGCTCATCTCCGAGATCACGCAG

228   Y  P  H  D  P  Y  K  V  I  V  N  D  Y  A  L  C  L  A  G  F
885   TACCCGCACGACCCGTACAAGGTGATTGTCAACGACTACGCGCTGTGCCTGGCCGGGTTC

243   V  G  A  I  S  A  Q  Q  P  W  G  D  L  A  H  F  V  S  C  L
945   GTTGGCGCCATCTCGGCGCAGCAGCCGTGGGGCGACCTCGCGCACTTCGTGTCGTGCCTG

258   C  F  S  Y  V  V  Y  S  L  W  S  C  F  T  G  A  I  D  G  E
1005  TGCTTCAGCTACGTCGTGTACTCGCTGTGGTCGTGCTTCACGGGCGCGATTGACGGCGAG

273   T  Q  C  N  V  E  K  S  G  L  R  W  I  R  F  S  T  I  T  T
1065  ACGCAGTGCAACGTCGAGAAGTCGGGCCTGCGGTGGATCCGGTTCTCGACCATCACGACG

288   W  S  L  F  P  I  T  W  F  S  Y  T  S  G  L  I  S  F  T  V
1125  TGGTCGCTGTTCCCCATCACGTGGTTCTCGTACACGTCGGGGCTCATCTCGTTCACGGTG

303   A  E  A  G  F  S  M  I  D  I  G  A  K  V  F  L  T  L  V  L
1185  GCCGAGGCCGGGTTCTCGATGATCGACATTGGCGCCAAGGTGTTCCTCACGCTGGTGCTG

318   V  N  S  T  V  E  Q  A  Q  N  Q  K  V  D  A  I  T  A  I  A
1245  GTCAACTCGACGGTCGAGCAGGCGCAGAACCAGAAGGTGGACGCCATCACGGCGATTGCC

333   E  E  L  E  N  Q  I  N  N  C  D  A  I  L  Q  K  M  M  P  E
1305  GAGGAGCTCGAGAACCAGATCAACAACTGCGACGCGATTCTGCAGAAGATGATGCCCGAG

348   G  V  L  E  Q  L  K  N  G  Q  A  T  E  A  K  E  Y  E  S  V
1365  GGCGTGCTCGAGCAGCTCAAGAACGGGCAGGCGACCGAGGCCAAGGAGTACGAGAGCGTG

363   T  V  F  F  S  D  I  T  N  F  T  V  I  S  S  R  T  S  T  K
1425  ACCGTCTTCTTCTCGGACATCACCAACTTCACGGTCATCTCGTCGCGCACCTCGACCAAG

378   D  M  M  A  T  L  N  K  L  W  L  E  Y  D  A  I  A  K  R  W
1485  GACATGATGGCGACGCTCAACAAGCTGTGGCTCGAGTACGACGCGATTGCCAAAAGGTGG

393   G  V  Y  K  V  E  T  I  G  D  A  Y  L  G  V  T  G  A  P  D
1545  GGCGTGTACAAGGTCGAGACGATTGGCGACGCGTACCTGGGCGTGACGGGCGCCCCCGAC

408   V  V  P  D  H  A  E  R  A  C  N  F  A  V  D  I  I  E  M  I
1605  GTGGTGCCCGACCACGCCGAGCGCGCGTGCAACTTTGCCGTGGACATCATCGAGATGATC

423   K  S  F  K  T  I  T  G  E  S  I  N  I  R  I  G  L  N  S  G
1665  AAGTCGTTCAAGACCATCACGGGCGAGTCCATCAACATCCGCATCGGGCTCAACTCGGGC

438   P  V  T  A  G  V  L  G  D  L  N  P  H  W  C  L  V  G  D  T
1725  CCCGTCACGGCCGGCGTGCTGGGCGACCTCAACCCGCACTGGTGCCTCGTCGGCGACACG

453   V  N  T  A  S  R  M  E  S  T  S  K  A  G  H  I  H  I  S  E
1785  GTCAACACGGCGTCGCGCATGGAGTCCACGTCCAAGGCCGGCCACATCCACATCTCCGAG

468   S  T  Y  H  F  I  K  S  K  F  V  T  Q  P  L  D  V  M  E  V
1845  TCGACGTACCACTTCATCAAGTCCAAGTTTGTCACGCAGCCGCTCGACGTCATGGAGGTC

483   K  G  K  G  K  M  Q  T  Y  W  V  L  G  R  K  *
1905  AAGGGCAAGGGCAAGATGCAGACGTACTGGGTGCTGGGCCGCAAGTAG
```

Figure S1. Nucleotide and deduced amino acid sequence of BeGC1 gene, Related to Figure 2. The arrow indicates the transcription start site determined in the 5´RACE assay. The rhodopsin domain (continuous line) and the guanylyl cyclase domain (dashed line) are underlined. The coiled-coil domain is overlined. In the Type I rhodopsin domain residues shown in red are conserved and make contact with retinal; the lysine that binds covalently to retinal (K255) is shown in cyan. In the guanylyl cyclase domain residues shown in green are important for catalysis and those in yellow recognize the guanine base in GTP.

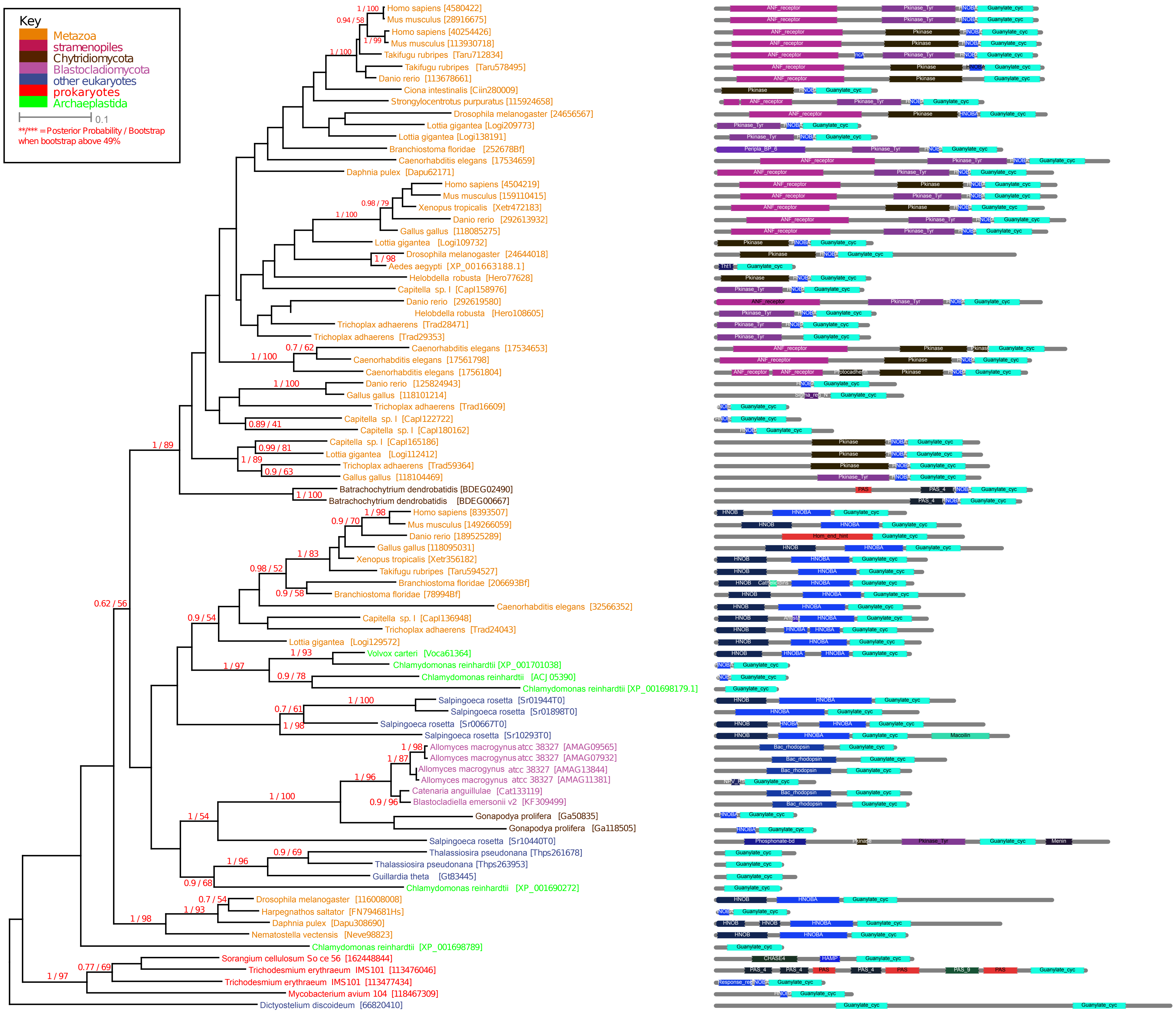

Key
Metazoa
stramenopiles
Chytridiomycota
Blastocladiomycota
other eukaryotes
prokaryotes
Archaeplastida
0.1
/* = Posterior Probability / Bootstrap when bootstrap above 49%
Homo sapiens [4580422]
Mus musculus [28916675]
Homo sapiens [40254426]
Mus musculus [113930718]
Takifugu rubripes [Taru712834]
Takifugu rubripes [Taru578495]
Danio rerio [113678661]
Ciona intestinalis [Ciin280009]
Strongylocentrotus purpuratus [115924658]
Drosophila melanogaster [24656567]
Lottia gigantea [Logi209773]
Lottia gigantea [Logi138191]
Branchiostoma floridae [252678Bf]
Caenorhabditis elegans [17534659]
Daphnia pulex [Dapu62171]
Homo sapiens [4504219]
Mus musculus [159110415]
Xenopus tropicalis [Xetr472183]
Danio rerio [292613932]
Gallus gallus [118085275]
Lottia gigantea [Logi109732]
Drosophila melanogaster [24644018]
Aedes aegypti [XP_001663188.1]
Helobdella robusta [Hero77628]
Capitella sp. I [Capl158976]
Danio rerio [292619580]
Helobdella robusta [Hero108605]
Trichoplax adhaerens [Trad28471]
Trichoplax adhaerens [Trad29353]
Caenorhabditis elegans [17534653]
Caenorhabditis elegans [17561798]
Caenorhabditis elegans [17561804]
Danio rerio [125824943]
Gallus gallus [118101214]
Trichoplax adhaerens [Trad16609]
Capitella sp. I [Capl122722]
Capitella sp. I [Capl180162]
Capitella sp. I [Capl165186]
Lottia gigantea [Logi112412]
Trichoplax adhaerens [Trad59364]
Gallus gallus [118104469]
Batrachochytrium dendrobatidis [BDEG02490]
Batrachochytrium dendrobatidis [BDEG00667]
Homo sapiens [8393507]
Mus musculus [149266059]
Danio rerio [189525289]
Gallus gallus [118095031]
Xenopus tropicalis [Xetr356182]
Takifugu rubripes [Taru594527]
Branchiostoma floridae [206693Bf]
Branchiostoma floridae [78994Bf]
Caenorhabditis elegans [32566352]
Capitella sp. I [Capl136948]
Trichoplax adhaerens [Trad24043]
Lottia gigantea [Logi129572]
Volvox carteri [Voca61364]
Chlamydomonas reinhardtii [XP_001701038]
Chlamydomonas reinhardtii [ACJ 05390]
Chlamydomonas reinhardtii [XP_001698179.1]
Salpingoeca rosetta [Sr01944T0]
Salpingoeca rosetta [Sr01898T0]
Salpingoeca rosetta [Sr00667T0]
Salpingoeca rosetta [Sr10293T0]
Allomyces macrogynus atcc 38327 [AMAG09565]
Allomyces macrogynus atcc 38327 [AMAG07932]
Allomyces macrogynus atcc 38327 [AMAG13844]
Allomyces macrogynus atcc 38327 [AMAG11381]
Catenaria anguillulae [Cat133119]
Blastocladiella emersonii v2 [KF309499]
Gonapodya prolifera [Ga50835]
Gonapodya prolifera [Ga118505]
Salpingoeca rosetta [Sr10440T0]
Thalassiosira pseudonana [Thps261678]
Thalassiosira pseudonana [Thps263953]
Guillardia theta [Gt83445]
Chlamydomonas reinhardtii [XP_001690272]
Drosophila melanogaster [116008008]
Harpegnathos saltator [FN794681Hs]
Daphnia pulex [Dapu308690]
Nematostella vectensis [Neve98823]
Chlamydomonas reinhardtii [XP_001698789]
Sorangium cellulosum So ce 56 [162448844]
Trichodesmium erythraeum IMS101 [113476046]
Trichodesmium erythraeum IMS101 [113477434]
Mycobacterium avium 104 [118467309]
Dictyostelium discoideum [66820410]

Figure S2. Phylogeny of the guanylyl-cyclase domain indicating a gene-fusion in the Blastocladiomycota fungi with a Type I rhodopsin domain, Related to Figure 2. Topology shown is a MrBayes phylogeny (100,000,000 generations, WAG+Γ, and a burn-in of 3,172 samples [sampled every 1,000 generations]). When consistent with the MrBayes topology and 50% or more bootstrap support values are added (from analysis in RAxML (LG + Γ) with 1,000 bootstraps). Support values in red: Bayesian posterior probability/ML bootstrap. Each branch is annotated with the PFAM domain architecture of the source sequence. The gene phylogeny includes a wider sampling of gene paralogues recovered from the sample dataset (Table S1) demonstrating cases of differential loss (for example in the 'chytrid' fungi sampled) and alternative protein domain architectures across this gene family as identified using PFAM [S1] All sequences are labelled with GenBank Accession numbers or genome sequencing centre Gene IDs. Sequences used in the alignment are available at https://github.com/guyleonard/blastocladiella/tree/master/publication/supplementary.

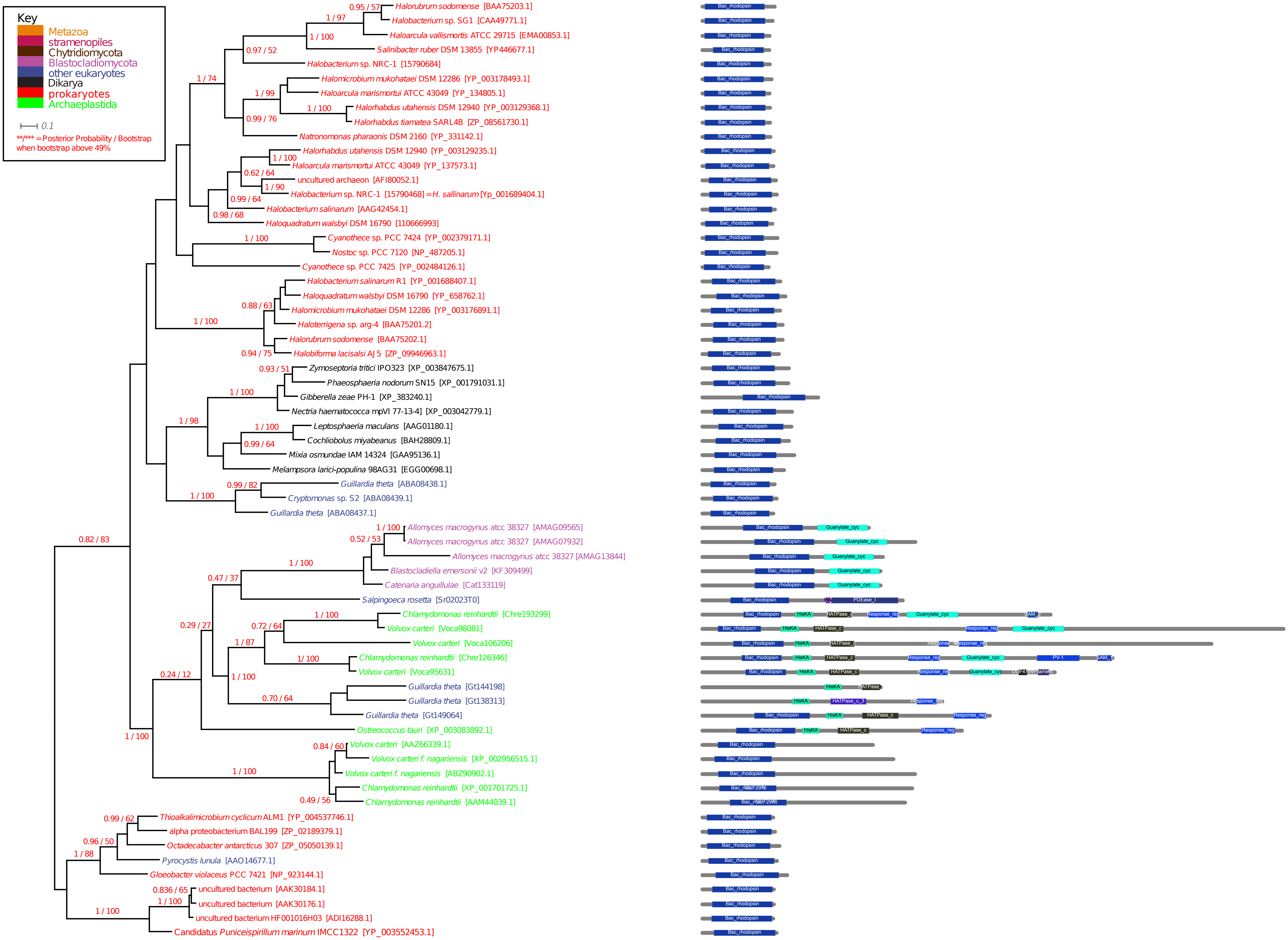
Key
Metazoa
stramenopiles
Chytridiomycota
Blastocladiomycota
other eukaryotes
Dikarya
prokaryotes
Archaeplastida
0.1
/* =Posterior Probability / Bootstrap
when bootstrap above 49%
0.95 / 57
Halorubrum sodomense [BAA75203.1]
1 / 97
Halobacterium sp. SG1 [CAA49771.1]
1 / 100
Haloarcula vallismortis ATCC 29715 [EMA00853.1]
0.97 / 52
Salinibacter ruber DSM 13855 [YP446677.1]
Halobacterium sp. NRC-1 [15790684]
1 / 74
Halomicrobium mukohataei DSM 12286 [YP_003178493.1]
1 / 99
Haloarcula marismortui ATCC 43049 [YP_134805.1]
1 / 100
Halorhabdus utahensis DSM 12940 [YP_003129368.1]
0.99 / 76
Halorhabdus tiamatea SARL4B [ZP_08561730.1]
Natronomonas pharaonis DSM 2160 [YP_331142.1]
Halorhabdus utahensis DSM 12940 [YP_003129235.1]
1 / 100
Haloarcula marismortui ATCC 43049 [YP_137573.1]
0.62 / 64
uncultured archaeon [AFI80052.1]
1 / 90
Halobacterium sp. NRC-1 [15790468] =H. sallinarum [Yp_001689404.1]
0.99 / 64
Halobacterium salinarum [AAG42454.1]
0.98 / 68
Haloquadratum walsbyi DSM 16790 [110666993]
1 / 100
Cyanothece sp. PCC 7424 [YP_002379171.1]
Nostoc sp. PCC 7120 [NP_487205.1]
Cyanothece sp. PCC 7425 [YP_002484126.1]
Halobacterium salinarum R1 [YP_001688407.1]
Haloquadratum walsbyi DSM 16790 [YP_658762.1]
0.88 / 63
Halomicrobium mukohataei DSM 12286 [YP_003176891.1]
1 / 100
Haloterrigena sp. arg-4 [BAA75201.2]
Halorubrum sodomense [BAA75202.1]
0.94 / 75
Halobiforma lacisalsi AJ 5 [ZP_09946963.1]
0.93 / 51
Zymoseptoria tritici IPO323 [XP_003847675.1]
Phaeosphaeria nodorum SN15 [XP_001791031.1]
1 / 100
Gibberella zeae PH-1 [XP_383240.1]
Nectria haematococca mpVI 77-13-4] [XP_003042779.1]
1 / 98
1 / 100
Leptosphaeria maculans [AAG01180.1]
0.99 / 64
Cochliobolus miyabeanus [BAH28809.1]
Mixia osmundae IAM 14324 [GAA95136.1]
Melampsora larici-populina 98AG31 [EGG00698.1]
0.99 / 82
Guillardia theta [ABA08438.1]
1 / 100
Cryptomonas sp. S2 [ABA08439.1]
Guillardia theta [ABA08437.1]
0.82 / 83
1 / 100
Allomyces macrogynus atcc 38327 [AMAG09565]
0.52 / 53
Allomyces macrogynus atcc 38327 [AMAG07932]
Allomyces macrogynus atcc 38327 [AMAG13844]
1 / 100
Blastocladiella emersonii v2 [KF309499]
0.47 / 37
Catenaria anguillulae [Cat133119]
Salpingoeca rosetta [Sr02023T0]
1 / 100
Chlamydomonas reinhardtii [Chre193299]
0.29 / 27
0.72 / 64
Volvox carteri [Voca98081]
1 / 87
Volvox carteri [Voca106206]
1 / 100
Chlamydomonas reinhardtii [Chre126346]
0.24 / 12
1 / 100
Volvox carteri [Voca95631]
Guillardia theta [Gt144198]
0.70 / 64
Guillardia theta [Gt138313]
Guillardia theta [Gt149064]
Ostreococcus tauri [XP_003083892.1]
1 / 100
0.84 / 60
Volvox carteri [AAZ66339.1]
Volvox carteri f. nagariensis [XP_002956515.1]
1 / 100
Volvox carteri f. nagariensis [ABZ90902.1]
Chlamydomonas reinhardtii [XP_001701725.1]
0.49 / 56
Chlamydomonas reinhardtii [AAM44039.1]
0.99 / 62
Thioalkalimicrobium cyclicum ALM1 [YP_004537746.1]
alpha proteobacterium BAL199 [ZP_02189379.1]
0.96 / 50
Octadecabacter antarcticus 307 [ZP_05050139.1]
1 / 88
Pyrocystis lunula [AAO14677.1]
Gloeobacter violaceus PCC 7421 [NP_923144.1]
0.836 / 65
uncultured bacterium [AAK30184.1]
1 / 100
uncultured bacterium [AAK30176.1]
1 / 100
uncultured bacterium HF001016H03 [ADI16288.1]
Candidatus Puniceispirillum marinum IMCC1322 [YP_003552453.1]
Bac_rhodopsin
Guanylate_cyc
PDEase_I
HisKA
HATPase_c
Response_reg
SAM_2
PV-1
HATPase_c_3
DUF2976

Figure S3. Phylogeny of the putative Type I rhodopsin domain indicating a gene-fusion in the Blastocladiomycota fungi with a guanylyl-cyclase domain, Related to Figure 2. Topology shown is derived from a MrBayes analysis (100,000,000 generations, calculated using a WAG+Γ substitution matrix, and a burn-in of 5,957 samples [sampled every 1,000 generations]). When consistent with the MrBayes topology and 50% or more bootstrap support values are added. Bootstrap analysis was conducted using RAxML (LG+Γ substitution matrix) with 1,000 bootstraps). Support values are in red: Bayesian posterior probability/ML bootstrap. Each branch is annotated with the PFAM domain architecture of the source sequence. This analysis also showed a unique rhodopsin gene fusion architecture in the green algae that involves a histidine kinase, a response regulator and a putative adenylyl/guanylyl-cyclase domain. Based on the fusion architecture and the results of this phylogeny and the Blastocladiomycota guanylyl-cyclase domain phylogeny (Figure S2), we suggest that this is a convergent fusion. All sequences are labelled with GenBank Accession numbers or genome sequencing centre Gene IDs. Sequences used in the alignment are available at https://github.com/guyleonard/blastocladiella/tree/master/publication/supplementary.

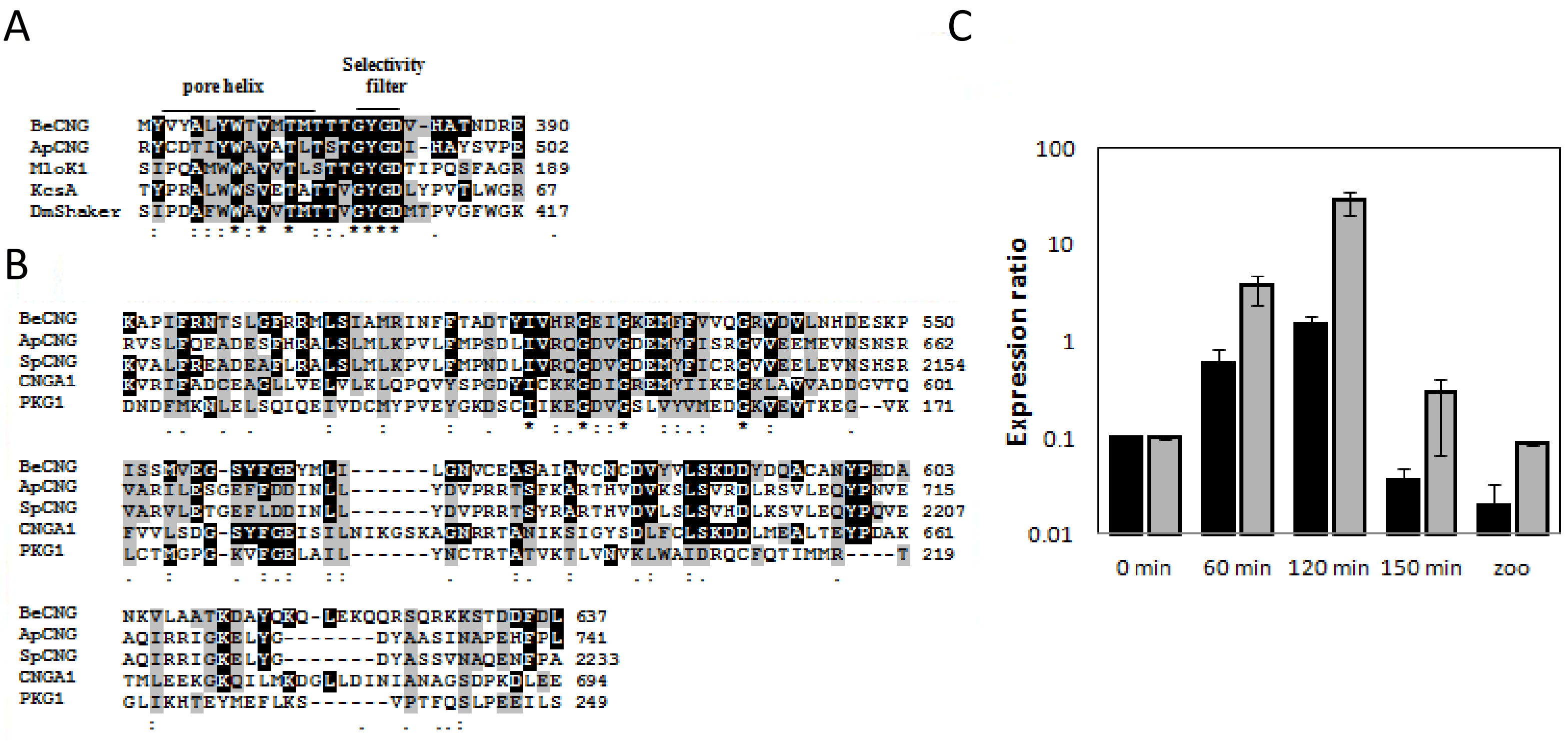

Figure S4 - Putative cyclic nucleotide gated channel BeCNG1: comparison of conserved sequence motifs (A and B) and transcript levels during *B. emersonii* sporulation, Related to Figure 1. (C). The sequences of the putative pore helix and selection filter are shown in **(A);** the cyclic nucleotide-binding domain is shown in **(B)**. The alignments include BeCNG1 (NCBI accession: KF309500); ApCNG, the cNMP gated potassium channel *Arbacia punctulata* (NCBI accession: ABH10136.1), MloK1, K+-selective CNG channel from *Mesorhizobium loti* (NCBI accession: NP_104392.1), KcsA, K+ channel from *Streptomyces lividans* (NCBI accession: Z37969.1), DmShaker, K+ channel from Drosophila (NCBI accession: X07131.1); SpCNG K+ channel from *Strongylocentrotus purpuratus* (NCBI accession: NM_001081964.1); CNGA1, cGMP-gated cation channel alpha-1 isoform 1 from *Homo sapiens* (NCBI accession: NP_001136036.1); PKG1, cGMP-binding site of PRKG1 (NCBI accession: NP_776703.1). Identical residues are marked with an asterisk (*), high conservation residues are marked with colons (:) and lower conservation residues are marked with a dot (.). The BeCNG channel was taken as reference. Residues identical to the reference are shown in black and conserved residues are shown in grey. **(C)** Transcript levels of *B. emersonii* BeCNG1 gene (black rectangles) evaluated by real-time RT-PCR at different times after induction of sporulation (0, 60, 120 and 150 min) and in the resulting zoospores (zoo). As a control, BeGC1 transcript levels (grey rectangles) were also analyzed. Data are mean values from three independent biological samples. Vertical bars represent standard deviation.

Supplemental Experimental Procedures

Genome sequencing of *Blastocladiella emersonii*.

Total DNA of a *Blastocladiella emersonii* isolate (ATCC 22665) was extracted from $4x10^9$ zoospores, as previously described [S2]. DNA was checked for both prokaryotic and eukaryotic contamination using general 18S and 16S SSU rDNA PCR. Each 50 µL PCR reactions contained 2 µL of each primer (10 $pM\mu L^{-1}$), 25 µL of Master Mix (Promega, containing 3 mM $MgCl_2$, 400 µM of each dNTP, and 50 U/mL of Taq DNA polymerase), 19 µL of PCR water, and 2 µL of a 1/1000 dilution of template DNA. Universal eukaryotic SSU rRNA primers 1F (5'CTGGTTGATCCTGCCAG-3') and 1520R (5'-CTGCAGGTTCACCTA-3') with the following cycling conditions were used; initial denaturation at 95 °C for 5 min, followed by 30 cycles of 95 °C for 1 min, 57 °C for 1 min, and 72 °C for 1.5 min, with a final extension at 72 °C for 10 min [S3]. For universal prokaryotic SSU rRNA amplification primers PA (5'-AGAGTTTGATCCTGGCTCAG-3') and PH (5'-AAGGAGGTCATCCAGCCGCA-3') [S4] with the following cycling conditions used, with *Escherichia coli* DNA acting as a positive control; initial denaturation of 94 °C for 5 min, followed by 30 cycles of 94 °C for 1 min, 55 °C for 1 min, 72 °C for 2 min, with a final step of 72 °C for 10 min.

The prokaryotic PCR was negative while the eukaryotic SSU PCR resulted in a single band of appropriate size. The 1F-1520R PCR reaction was purified using the Wizard ® SV gel and PCR clean-up system (Promega) and sequenced directly on both strands by Cogenics (Essex). Chromatograms were checked for inconsistencies, which could be the result of a multi-template amplification and the derived sequences used for BLASTn

(GenBank nr database) demonstrating the DNA was derived from a pure culture of the target microbe.

We sequenced the *Blastocladiella* genome using two approaches: 1) Two 454 FLX titanium (Roche) paired-end libraries, one at 3kb and the other at 20kb, and 2) Illumina GA2 paired-end 76nt sequencing. The DNA was prepared for both sequencing platforms using the standard protocols. The 454 approach generated 1,099,679 sequence reads resulting in 345,422,838 bp of sequences for the 3kb library and 189,318 sequence reads resulting in 67,116,270 bp of sequences for the 20kb library. Two lanes of Illumina GA2 paired-end 76bp sequencing was performed yielding a total of 41,083,984 reads. A total of 3,535,030,995 bp from all sequencing technologies. Quality filtering for the Illumina reads was achieved by removing bases 1-10 and 62-76.

Several assembly approaches were tested but finally the *Blastocladiella* genome sequence was assembled using a hybrid approach similar to that of the Fire Ant genome [S5]. Firstly the Velvet assembler (1.0.09) [S6] was used with the Illumina data and resulted in 37,387 contigs spanning 25,868,447bp at a mean coverage of 52x. Kmer size of 33 was used along with expected kmer coverage of 15 and a coverage cutoff of 0.9 as determined by the Velvet Optimiser 2.14 script bundled with Velvet. This produced an N50 contig-length of 2,594 bp. The EMBOSS tool 'splitter' was used to split up the Illumina-based contigs into pseudo-reads that were 400bp long with an overlap of 200p. The pseudo-reads were then used with the Roche 454 assembler 'newbler' (http://www.454.com/products/analysis-software/) also known as 'GS De Novo Assembler' version 2.5 with standard settings, along with the three 'sff' – flow gram – files from the 454 sequencing (total of 423,386,588 bp). This resulted in 14,099 contigs with an N50 of

3,912bp and an L50 of 52. Newbler also produced 772 scaffolds with an N50 of 155,559 bp. The estimated genome size is 60.4Mb and our total coverage estimate is 58.5x. CEGMA analysis [S7] demonstrated 19 partial genes missing (7 of which are missing from all three publically available Blastocladiomycota genome assemblies) from a list of 248 'core' genes suggesting the genome assembly contains 95% of the predicted proteome.

Open reading frames for predicted proteins were identified using the MAKER pipeline [S8], which combined several gene-calling methods (MAKER, SNAP (http://korflab.ucdavis.edu/software.html) and Augustus [S9] and the *Blastocladiella* ESTs [S10] as a reference dataset (which had been manually checked to remove potential sources of contamination). Several iterations of the pipeline were carried out, similarly to the "MAKER Tutorial 2012" (http://gmod.org/wiki/MAKER_Tutorial_2012) help pages. Augustus was run using the reference genomes of *Rhizopus oryzae*, *Neurospora crassa*, *Ustilago maydis* and *Saccharomyces cerevisiae*. The output from these runs, the SNAP runs and MAKER were combined, de-duplicated, and clustered using the program CD-HIT with a 0.95% identity. The longest ORF for each cluster was picked as the representative of that cluster, resulting in 23,790 predicted ORFs for *Blastocladiella emersonii*. Each ORF was then BLAST searched locally against a database of genomes (see Table S1). All contigs that did not return a top hit that was not an opisthokont were manually inspected for evidence that they were contaminating sequences. These searches did not identify contaminant sequence reads.

Gene phylogenies

Each of the three individual domains derived from two genes (BeGC1: guanylyl cyclase enzyme domain and rhodopsin-like domain [GenBank Accession no. KF309499] and

BeCNG: putative K^+-selective channel [GenBank Accession no. KF309500) were used as seed sequence for a custom bioinformatic pipeline for generating phylogenies [S11], which uses BLASTp to recover a set of amino acid sequences (gathering threshold set to 1e-10) from a local database of published and publicly available genome databases. (Table S1). The retrieved sequences are then aligned using MAFFT v7.03b [S12] and then masked using the program TrimAl [S13] (to remove gaps and highly variant sites) and a preliminary tree calculated using FastTree [S14] with aLRT topology support values. These trees and alignments are used as guides for manual improved taxon sampling, sequence alignment, and alignment masking. Alignments were confined to sequences that we could achieve a reasonable gene alignment, in many cases minimizing sampling of the wider gene family (e.g. rhodopsin). For each dataset, long branches and closely related sequences from the same genus groups were removed and additional taxa sampled (using manual BLAST searching of NCBI nr database, Broad genome database and JGI Genome Portal). Once the set of sequences were finalised they were realigned with MAFFT and manually masked. These alignments were then passed to the program ProtTest3 [S15] to predict the 'best-fit' model of sequence substitution for phylogenetic analysis (details of the model are given in the figure legends that accompany each phylogeny). The settings predicted by ProtTest are then used - where possible - with the programs RAxML [S16] to generate a ML tree, (100 BKL and 1,000 BS) under the CAT model and with MrBayes3.2 (until the logLikelihood reaches a plateau for a minimum of 500,000 generation samples (sampled every 1,000 generations) – burnin calculated using Tracer v1.5 [S17]. Downstream scripts were used to produce annotated phylogenies with PFAM domains [1], multiple bootstrap/posterior probabilities and taxonomic classifications.

Phototaxis assay in growth media agar plates

Vegetative cells growing on PYG agar plates (0.13% peptone, 0.13% yeast extract, 0.3% glucose, and 1.5% agar) in the dark for 16h at 19°C were induced to sporulate also in the dark, by adding 2 ml of sterile water. The released zoospores were collected, filtered through nitex cloth, diluted in sterile water at a density of 2x10^{4}/ml. After maintaining the zoospores for 30 min in the dark they were inoculated in PYG agar plates to which 1 ml of sterile distilled water was previously added, to permit the zoospores to swim. Zoospores (50 µl) were carefully deposited on the plate in a position diametrically opposite to where the light beam was to be applied. The plates were immediately inserted individually inside black plastic envelopes. In the envelope that contained the plate to be exposed to the light beam, a hole of 0.5 cm in diameter was made in the plastic envelope in a position diametrically opposite to the point where the zoospores were inoculated. In the control plates no hole was made in the plastic envelope. Home-built LED light sources of 522 ± 17 nm wavelength and 4.4 mW/cm^{2} intensity or 633 ± 13 nm wavelength and 4.4 mW/cm^{2} intensity were applied over the plates, which were incubated at room temperature for 4h to allow the zoospores to germinate and enter vegetative growth. After further incubation at 17°C overnight, the plates were observed under a light microscope. The number of cells in the region of the light beam were counted and photographed. In the control plates, the corresponding region was also photographed and the number of cells counted. Phototaxis assays were also carried out after photobleaching of rhodopsin, by pre-incubating zoospores with hydroxylamine (500 µM) and green light (522 ± 17 nm wavelength; 4.4 mW/cm^{2} intensity) for 10 min, and then repeating the procedure described above.

Phototaxis in the microfluidic chamber

Zoospores ($2x10^4$/ml) obtained as described in phototaxis in agar plates were kept in the dark for 30 min and then injected in an opaque microfluidic chamber containing a transparent area at the opposite end relative to the injection channel. Cells were observed with an inverted light microscope in a dark room to reduce environmental noise. Chamber dimensions were 100 µm in height, 1mm wide and 4cm long [S18]. The initial cell number was recorded at the transparent zone and cells were allowed to swim freely for 10 minutes, when cells were once again counted. The microscope light, which was used at three different wavelengths: green light (565 ± 25 nm; 55 µW/cm^2); blue light (465 ± 25 nm; 67 µW/cm^2) or red light (620 ± 30 nm; 35 µW/cm^2), was kept on during the 10 minutes as the "light" condition and kept off for the "dark" condition. The experiments with retinal analogs were carried out with zoospores obtained growing the fungus in the presence of the carotenogenesis inhibitor norflurazon (50 µM) for three generations, and then incubating the zoospores in the dark for 40 min with 5 µM of either retinalA1 or retinalA2 prior to phototaxis. Four independent biological replicas were performed for each condition and cell counts were normalized according to cell density. Statistical significance was assessed by a Student's T test with p-value cutoff of 0.05. Phototaxis experiments were also carried out in the presence of 10 µM of the GC inhibitor LY83583 [S19].

Determination of intracellular levels of cGMP

Zoospores obtained as described in phototaxis assays were collected, filtered through nitex cloth and diluted in sterile water at a density of $8x10^6$/ml. The suspension containing the zoospores was kept in the dark for at least 30 min before being exposed to a home-built

LED light source of wavelength of 522 ± 17 nm and 4.4 mW/cm^2 of intensity. After different times, aliquots of 0.9 ml were transferred to an eppendorf tube and used for determination of the amount of cGMP present in the sample. For that, the zoospores were lysed by adding 100 µl of 1M HCl and total cell extracts were immediately frozen in dry ice. The amount of cGMP was determined using the EIA Direct cyclic GMP kit (Sigma), which is a competitive immunoassay for the quantitative determination of cGMP. Changes in cGMP levels in zoospores exposed to green light were also determined after photobleaching of rhodopsin by pre-incubating zoospores with hydroxylamine (500 µM) and green light (522 ± 17 nm and 4.4 mW/cm^2 of intensity) for 10 min, followed by a 30 min-incubation in the dark. The zoospore suspension was then exposed to green light, and aliquots were collected at different time points for cGMP determination. All assays were made in biological triplicates. Levels of cGMP were also determined in experiments where endogenous retinal was substituted by retinalA1 using zoospores obtained from growth in the presence of the carotenogenesis inhibitor norflurazon (50 µM) for three generations, and then incubating or not with 5 µM of retinalA1 for 40 min in the dark prior to exposure to green light (522 ± 17 nm; 4.4 mW/cm^2) for different times.

Western Blot

Total zoospore extracts were prepared by freezing and thawing of the cells. Unbroken zoospores were separated by centrifugation of the cell lysate for 5 min at 1,000 x g. The supernatant was then fractionated by centrifugation of the cell extracts for 5 min at 12,000 x g. The resulting pellet (12,000 ppt) was saved, and the supernatant was further centrifuged at 100,000 x g for 10 min. All centrifugation steps were carried out at 4^oC. The resulting pellet (100,000 ppt) and supernatant (100,000 sup) were saved and all fractions were

analyzed by Western blotting. Equal amounts of protein from each fraction were separated by denaturing SDS-polyacrylamide gel electrophoresis, and the resolved proteins were transferred to nitrocellulose filter. Membranes were incubated overnight at 4°C with 1:1,000 dilution of anti-BeGC1 in Tris-HCl, pH 8.0, containing 150 mM NaCl, 0.002% Tween 20, and 0.03% Triton X-100. The blots were developed using the secondary antibody CF680 Goat Anti-rabbit IgG (Uniscience) at a 1:10,000 dilution. The bound complex was detected using the Odyssey Infrared Imaging System (Li-Cor Biosciences; Lincoln, NE). The antiserum against BeGC1 was obtained from a rabbit immunized with a recombinant polypeptide expressed in *E. coli* corresponding to the GC catalytic domain of BeGC1 fused to a histidine-tag at the N-terminus. As a control, we used an antiserum against BePAT1, a cytoplasmic membrane bound ATPase from *B. emersonii* [S20], and anti-α-tubulin (Sigma).

Immunofluorescence microscopy

Zoospores were collected by centrifugation at 1000 x g for 5 min and fixed with 4% p-formaldehyde, 1% calcium chloride for 30 min. After, the cells were permeabilized with PBS containing 0.1% Triton X-100, blocked with 1% BSA and incubated at 37°C for 30 min with a rabbit anti-BeGC1 antiserum (1:50) The reactivity was developed with specific Goat anti-rabbit IgG antibodies conjugated to Alexa-Fluor 488 (Molecular Probes). For the visualization of the lipid droplets of the eyespot, a lipid-specific fluorescent dye Nile Red (Invitrogen) was added to the zoospore suspension at a dilution of 1:10,000. Images taken in a Nikon Eclipse E600 microscope were obtained under identical settings and were submitted to deconvolution with Huygens Essential image processing program.

Rapid Amplification of cDNA Ends (5’RACE)

Rapid amplification of the 5’ cDNA end (5’RACE) was performed according to instructions from the GeneRacer™ RACE Ready cDNA Kit (Invitrogen), using the primers included in the kit and gene specific primers (GSPs) designed based on expressed sequence tags (GSP1GC1: 5´CGAGAACCGGATCCACCGCAGGCCCG3´ and GSP2GC1: 5´ATGAAGTTGACGCCGAGGAGGCACAGC3´) [S10]. RACE products from the PCR were cloned in pGEM®-T vector system (Promega) and sequenced.

Real-time reverse transcription-PCR (qRT-PCR)

Five micrograms of total RNA were reverse transcribed using 200 U of SuperScriptIII reverse transcriptase (Invitrogen) and 500 ng of random nonamers according to manufacturer’s instructions. Approximately 180 ng of the resulting cDNA were used as template in the PCR assay, which included 800 nM of the forward and reverse primers and 10 µl of Platinum SYBR Green qPCR SuperMix UDG (Applied Biosystems). Primers used were as follows: BeGC′1, 5 -GACATCTGGTACGGGTACGG-3′ (forward) and 5′-GGAGATGAGCAGGATGAGGA-3′ (reverse), BeCNG, 5′-GAATTCCCTGACGTGGTGGGACTACT-3′ (forward) and 5′-GCGGCCGCTCTCGAACAGGTCAAAGTCG-3′ (reverse). Quantitative real time RT-PCR experiments (qRT-PCR) were performed using the GeneAmp 5700 Sequence Detection System (Applied Biosystems) equipment and the thermocycling conditions comprised an initial step at 50 °C for 2 min, followed by 95 °C for 10 min, 40 cycles of 95 °C for 15 s, and 60 °C for 1 min. For each gene and sporulation point analyzed, three independent RNA samples were used. The gene encoding the mitochondrial RNA helicase-

like protein was used as the calibrator gene in all experiments. Determination of the expression ratios was carried out using the $\mathbf{2^{-\Delta\Delta CT}}$ method [S21].

S1. Bateman, A., Coin, L., Durbin, R., Finn, R.D., Hollich, V., Griffiths-Jones, S., Khanna, A., Marshall, M., Moxon, S., Sonnhammer, E.L., et al. (2004). The Pfam protein families database. Nucleic Acids Res *32*, D138-141.

S2. Marques Mdo, V., and Gomes, S.L. (1992). Cloning and structural analysis of the gene for the regulatory subunit of cAMP-dependent protein kinase in *Blastocladiella emersonii*. J Biol Chem *267*, 17201-17207.

S3. Lefevre, E., Bardot, C., Noel, C., Carrias, J.F., Viscogliosi, E., Amblard, C., and Sime-Ngando, T. (2007). Unveiling fungal zooflagellates as members of freshwater picoeukaryotes: evidence from a molecular diversity study in a deep meromictic lake. Environ Microbiol *9*, 61-71.

S4. Edwards, U., Rogall, T., Blocker, H., Emde, M., and Bottger, E.C. (1989). Isolation and direct complete nucleotide determination of entire genes. Characterization of a gene coding for 16S ribosomal RNA. Nucleic Acids Res *17*, 7843-7853.

S5. Wurm, Y., Wang, J., Riba-Grognuz, O., Corona, M., Nygaard, S., Hunt, B.G., Ingram, K.K., Falquet, L., Nipitwattanaphon, M., Gotzek, D., et al. (2011). The genome of the fire ant Solenopsis invicta. Proc Natl Acad Sci U S A *108*, 5679-5684.

S6. Zerbino, D.R., and Birney, E. (2008). Velvet: algorithms for de novo short read assembly using de Bruijn graphs. Genome Res *18*, 821-829.

S7. Parra, G., Bradnam, K., Ning, Z., Keane, T., and Korf, I. (2009). Assessing the gene space in draft genomes. Nucleic Acids Res *37*, 289-297.

S8. Cantarel, B.L., Korf, I., Robb, S.M., Parra, G., Ross, E., Moore, B., Holt, C., Sanchez Alvarado, A., and Yandell, M. (2008). MAKER: an easy-to-use annotation pipeline designed for emerging model organism genomes. Genome Res *18*, 188-196.

S9. Stanke, M., and Morgenstern, B. (2005). AUGUSTUS: a web server for gene prediction in eukaryotes that allows user-defined constraints. Nucleic Acids Res *33*, W465-467.

S10. Ribichich, K.F., Salem-Izacc, S.M., Georg, R.C., Vencio, R.Z., Navarro, L.D., and Gomes, S.L. (2005). Gene discovery and expression profile analysis through sequencing of expressed sequence tags from different developmental stages of the chytridiomycete *Blastocladiella emersonii*. Eukaryot Cell *4*, 455-464.

S11. Richards, T.A., Soanes, D.M., Foster, P.G., Leonard, G., Thornton, C.R., and Talbot, N.J. (2009). Phylogenomic analysis demonstrates a pattern of rare and ancient horizontal gene transfer between plants and fungi. Plant Cell *21*, 1897-1911.

S12. Katoh, K., and Standley, D.M. (2013). MAFFT multiple sequence alignment software version 7: improvements in performance and usability. Mol Biol Evol *30*, 772-780.

S13. Capella-Gutierrez, S., Silla-Martinez, J.M., and Gabaldon, T. (2009). trimAl: a tool for automated alignment trimming in large-scale phylogenetic analyses. Bioinformatics *25*, 1972-1973.

S14. Price, M.N., Dehal, P.S., and Arkin, A.P. (2010). FastTree 2--approximately maximum-likelihood trees for large alignments. PLoS One *5*, e9490.

S15. Darriba, D., Taboada, G.L., Doallo, R., and Posada, D. (2011). ProtTest 3: fast selection of best-fit models of protein evolution. Bioinformatics *27*, 1164-1165.

S16. Stamatakis, A. (2006). RAxML-VI-HPC: maximum likelihood-based phylogenetic analyses with thousands of taxa and mixed models. Bioinformatics *22*, 2688-2690.

S17. Rambaut, A.D., A.J. (2007). Tracer v1.5 [computer program]. Avaliable: http://beast.bio.ed.ac.uk/Tracer.

S18. Meng, Q.H., Zhou, L.X., Luo, J.L., Cao, J.P., Tong, J., and Fan, S.J. (2005). Effect of 7-hydroxystaurosporine on glioblastoma cell invasion and migration. Acta Pharmacol Sin *26*, 492-499.

S19. Schmidt, M.J., Sawyer, B.D., Truex, L.L., Marshall, W.S., and Fleisch, J.H. (1985). LY83583: an agent that lowers intracellular levels of cyclic guanosine 3',5'-monophosphate. J Pharmacol Exp Ther *232*, 764-769.

S20. Fietto, L.G., Pugliese, L., and Gomes, S.L. (2002). Characterization and expression of two genes encoding isoforms of a putative Na, K-ATPase in the chytridiomycete *Blastocladiella emersonii*. Biochim Biophys Acta *1576*, 59-69.

S21. Livak, K.J., and Schmittgen, T.D. (2001). Analysis of relative gene expression data using real-time quantitative PCR and the 2(-Delta Delta C(T)) Method. Methods *25*, 402-408.
